# Dental age estimation: a scoping review comparing the manual application of the Demirjian method and artificial intelligence modalities

**DOI:** 10.1007/s00414-026-03721-4

**Published:** 2026-02-23

**Authors:** Stephanie Baylis, Joanna F. Dipnall, Richard Bassed

**Affiliations:** 1 Department of Forensic Medicine, School of Public Health and Preventive Medicine, Monash University, Melbourne, VIC Australia; 2 Baylis Dental Services, Whangarei, Northland, New Zealand; 3 School of Public Health and Preventive Medicine, Monash University , Melbourne, VIC Australia; 4 Institute for Mental and Physical Health and Clinical Translation, School of Medicine, Deakin University, Geelong, Australia; 5 Department of Forensic Medicine, Victorian Institute of Forensic Medicine, Monash University, Melbourne, VIC Australia

**Keywords:** Demirjian stages, Legal consequences, Black box, Human rights, Adult vs minor

## Abstract

The investigation of the viability using Artificial intelligence (AI) compared to the Demirjian method in dental age estimation is growing. This scoping review looked at how AI-assisted dental age estimation models compare to the Demirjian method and their usefulness to general and forensic dentists. Searches were conducted in Medline, Embase, Global Health, Scopus, Social Science Premium Collection, CENTRAL-Cochrane Library Central Register of Controlled Trials, and NZLII-New Zealand Legal Information Institute. Google and Google Scholar searches were also undertaken to identify any grey material. Twenty-two articles were included in this review, organised into three groups to assist comparisons i.e., stage allocation, stage allocation prior to AI model application, and use of the Demirjian method on study samples prior to applying AI models. Comparisons were difficult due to considerable variation in samples, dental features used, metrics, AI modelling, unstated regression methods, and reporting. Of the twenty-two studies included in this review, nine showed similar performance between experts manually applying Demirjian stages with/out scoring and AI models, four reported the AI models were better, two reported AI models were worse, four reported mixed results, one did not recommend either, while two did not report a comparison. Lessons learned from the traditional methods of dental age estimation have been ignored in many respects and important real-world requirements for legal and clinical applications are being overlooked.

**Supplementary Information:**

The online version contains supplementary material available at 10.1007/s00414-026-03721-4.

## Introduction

 Dental age estimation (DAE) is the comparative analysis of the developmental or regressive stages of an individual’s dentition, utilising relevant and validated reference data, to estimate the age of that individual. DAE is utilised by forensic practitioners to assess age of living or deceased individuals, assisting in the identification process, or safeguarding human rights [1–3]. Varying radiographic imaging modalities have been utilised e.g., orthopantograms (OPG/Panex), magnetic resonance imaging (MRI), or computed tomography (CT), to assess single or multiple dental features [4–13]: OPGs being the most frequently used due to accessibility. Recent DAE research has been focused on the application of Artificial Intelligence (AI) to reduce time, cost, error, subjective bias, and intra and inter-observer variability [14, 15].

AI encompasses machine, deep learning (DL), and generative AI with a view to imitating human intelligence by problem-solving through data classification [8, 16–19], age thresholding [18], or prediction [11, 13, 20, 21]. A systematic review by Niño‑Sandoval et al. explained how machine learning (ML) incorporated aspects of statistics, mathematical optimization, and data mining, for age determination [22]. Five common types of ML techniques include supervised [23, 24], unsupervised [6, 9, 25–27], semi-supervised [5, 7, 9, 28], transfer [9, 16–19, 29, 30], and reinforcement learning (refer to Online Resource 1. for definitions), although examples of reinforcement learning are not known in dental age estimation. Supervised machine learning may include linear or non-linear regression models (e.g. inclusion of polynomials, interactions) and can be regression or classification models. There are several parametric [10, 18, 23] and non-parametric [23, 24, 31–36] models used in dental age estimation (refer to Online Resource 1. for definitions). Artificial neural networks (ANNs), designed to mimic the human brain, are composed of neurons which can be adjusted or trained. These neurons are distributed in layers where the deeper the layer the more complex the ANN e.g., multilayer perceptrons (a perceptron is a building block) [22]. Deep learning involves ANNs e.g., convolutional neural networks (CNNs), where multilayer neural networks (interconnected layers of nodes) can be applied to both supervised and unsupervised models (i.e. no target labels) to recognise complex patterns with the ability to make decisions or predictions.

Several studies have compared the performance of automated methods to human experts with mixed results [22, 25, 28, 32, 37]. All methods require human intervention to some degree. However, the potential problem has been that any errors or bias introduced early on can affect the performance of AI outputs and hence validity and integrity [38].

There has been considerable inconsistency in the way many studies, traditional or AI assisted, have been conducted. Bias can occur, and compound, owing to several factors, such as data collection methods, populations, size and distribution of a dataset, age range, methods of analysis, selection of metrics used, and reporting of results [39]. Small sample size, inadequate sample description, uneven age distribution, different age ranges, have all affected model performance, hampered comparisons between groups and within the same population [25, 40, 41]. The statistical effect of small samples has been exacerbated by uneven age distribution, causing bias and variable error rates [42]. Brownlee and others explained the importance of employing large datasets when using ML, as small datasets may result in the model showing a high variance and overoptimistic performance [43]. Higher accuracies have been generally obtained in studies with larger sample sizes [44]. The large data sets required for DL models to train may be difficult to obtain [45, 46]. Training ANNs on small datasets may result in overfitting whereby the ML model learns well on the training set but poorly on any new/test data [4, 46]. According to Şahin et al. at least one thousand examples per class are necessary for AI image classification [46]. Calibration (training) of the human experts performing traditional DAE methods is also critical, although experienced dentists require smaller datasets on which to train, due to their experience in discerning differences and similarities within and between radiographs [47, 48]. Studies have shown that subjectivity is reduced and intra- and inter-observations are more reliable with increased levels of training and experience [49]. A study by Olze et al. found the Demirjian method to perform the best for observer agreement [50]. Dhanaj et al. compared intra- and inter-examiner observer agreements between four DAE methods and concluded the Demirjian method was preferred, having scored very good agreement for both types of observations [51].

Traditional regression methods used by manual DAE methods, have been found to overestimate the age of younger individuals and underestimate older individuals when applied to different groups, with the potential to incorrectly assess a minor as an adult, with potentially negative consequences [3, 52–54]. These modelling methods also introduce bias, known as age-mimicry or attraction to the middle i.e., age estimates are closer to or shift towards the mean age rather than the chronological age (CA). Furthermore, unless the target sample has a similar age distribution to the reference sample, the age estimates will be biased toward the reference sample distribution [54–57]. Ignoring maturity scoring to estimate chronological age and focusing on age of attaining each stage (i.e., mean age entering a stage), has been found to give more insight regarding variation between sexes, populations, and age groups. “Mean age entering a stage is the age at which half the individuals in the group have entered or passed the stage and is not the median aged child to enter the stage. This includes the most mature stage H “ [58]. Stage H is defined by Demirjian et al. [59], as a completely closed root apex with uniform periodontal membrane width around the apex. In many cases no distinction is made between mean age within stages or mean age entering stages. Sample size and age structure are important as if more than “half of the youngest group have reached or passed the stage, the sample age is too old to describe the average age entering the stage” [58]. Liversidge [40] and others, recommended transition analysis (TA) as an appropriate statistical method to compare timing of individual tooth stages, estimate age, and compare groups. Employing TA (probit or logistic regression) to estimate mean age entering stages gives more accurate estimations of age than traditional regression methods and reduce age-mimicry [40, 52, 57, 60–62].

The advancement of statistical methodologies has made the comparison of results with some traditional methods very difficult due to the utilisation of differing metrics e.g., standard deviations (SD), confidence intervals (CI), mean difference (MD) vs. mean absolute difference (MAD), etc [63, 64]. Niño-Sandoval et al. concluded that although there were advantages of ML in data processing and DL in data collection and analysis, non-comparable data was a limitation, with more information needed for comparison of the techniques. “Shan et al. only reported mean absolute error for the traditional methods but reported root mean square error (RMSE), mean square error (MSE), mean absolute error (MAE), and root squared (R^2^), for the ML methods [31]. Kumagai et al. did not report MAE for their Japanese sample but did for their Korean sample [32]. Shen et al. (2021 and 2022) did not report R^2^ for their manual methods. Zaborowicz et al. (2022) reported MAE but only reported ME and R^2^ for a few data” [22].

Multiple studies include sex-matched samples but do not report sex-specific results, while comparing themselves to traditional methods e.g., Demirjian et al., who analysed sex groups independently, showed distinct differences between sex. Sivri et al. were “unable to record sex for the sample due to security restraints” and yet estimated age using the Demirjian method. It is unclear whether they used the male or female weighted scores. They report their method could be used as an alternative to Demirjian et al. [65] whereas Koch et al. reported the Sivri technique was superior to Demirjian et al. [37].

To assist general and forensic practitioners DAE research needs to have real-world application, be reliable and reproducible. The increasing use of ML in DAE and heterogenous results from these techniques indicates there is a need to evaluate their viability. Thus, this scoping review aimed to understand whether AI can outperform humans and/or improve on manual methods of staging and/or age prediction using the Demirjian method of DAE utilising OPGs.

## Methods

### Protocol and registration

The protocol in this scoping review was designed using the Preferred Reporting Items for Systematic reviews and Meta-Analyses extension for Scoping Reviews (PRISMA-ScR). This review has not been assigned a registration number since PROSPERO currently does not include scoping reviews.

### Eligibility criteria

Eligibility criteria were set for the screening process as: not Demirjian/modified Demirjian studies; not OPG/Panoramic; not AI/ML/DL; not DAE; non-dental feature/s; not human. Exclusion criteria for full-text studies included sample bias, unclear sample distribution, comparator bias/exclusion, missing results, unclear methodology, indirect comparisons, irrelevant methodology, multiple papers with sample methodology by one research team. The following inclusion criteria were considered eligible: quantitative research articles; application of AI/ML/DL involving the Demirjian/modified Demirjian techniques; available in English language; full-text studies; OPG/Panoramic; study age-ranges included children/juveniles/subadults.

### Information sources

The following seven scholarly literature databases were searched on the 5th September 2024 and again; Medline (Ovid Platform), Embase (Ovid Platform), Global Health (Ovid Platform), Scopus (Elsevier Platform), Social Science Premium Collection (Proquest Platform), CENTRAL-Cochrane Library Central Register of Controlled Trials (Wiley Platform), and NZLII-New Zealand Legal Information Institute (NZLII Website). References cited in the selected studies and any systematic reviews found, were reviewed for further relevant studies. One study correction was sought. A search of Google and Google Scholar using the same keywords covering the main concepts of DAE and ML, was undertaken to locate any further relevant grey literature, on the 26th April 2025.

### Search

Search strategies were drafted by SB and refined through team discussion. With the assistance of a medical librarian, a combination of subject headings and keywords were searched using the Boolean Operator OR, covering the main concepts of DAE and ML. These two concepts were then combined with the Boolean Operator AND. No limits or filters were applied, and each database was searched from their inception date. A re-run of all databases to capture recent references was conducted on the 9th April 2025, with the publication limit of 5th September 2024-onwards placed for all databases. A detailed search strategy for each database listing all subject heading and keywords used for this search can be found in Online Resource 2.

### Selection of sources of evidence

The final search results were exported into Covidence Systematic Review software [66] for removal of duplicates and undertaking of the screening process. Two reviewers (SB and AF) screened titles and abstracts, within Covidence. Any conflicts were discussed and resolved with two other reviewers (JD and RB).

### Data charting process

Full text reading following of the selected articles was carried out by the SB with support JD and RB, to determine the articles for inclusion in this review. An Excel worksheet was used to extract relevant information from the articles.

### Data items

Data was entered under the following headings: Author; Year; Used Demirjian Scores(Sc)/Stages(St)/both; DAE method and number of stages; Study sample size; Study age range; Study population/s; Dental features; Sex(M/F/combined); AI algorithm architecture; Classification/DAE/both; Reported metrics; Comparison to experts(Y/N).

### Critical appraisal of individual sources of evidence

Each source of evidence was appraised in comparison to the traditional Demirjian method of assigning discrete dental development stages and applying weighted scores, if used. This included sample size, distribution, sex-matching, ethnicity, statistical methodology, rationales, and conclusions. The use of modified Demirjian methods and reported results were also evaluated in this way.

### Synthesis of results

The included articles were split into three groups with the aim of matching similar study approaches to undertake comparison of study methods, materials, sample distribution, reported metrics, and study results.

1. *Stage Allocation Alone*: Studies that employed AI algorithms to allocate Demirjian or modified Demirjian stages.
2. *Stage Allocation Prior to AI*: Studies that utilised Demirjian staging before progressing to age predictions using AI models.
3. *Demirjian Prior to AI*: Studies that applied the Demirjian scoring method to their specific populations before undertaking age predictions with various AI models.

Following the critical appraisal of the data, the following comparisons were undertaken within each group: the traditional Demirjian method versus modified methods; sample distributions including size, distribution, sex-matching; reported accuracy of the application of traditional Demirjian to ethnic group; image manipulation; reported metrics; performance of human experts and AI models; studies that made comments about how their AI models compared to traditional methods.

## Results

### Selection of sources of evidence

**Fig. 1:**
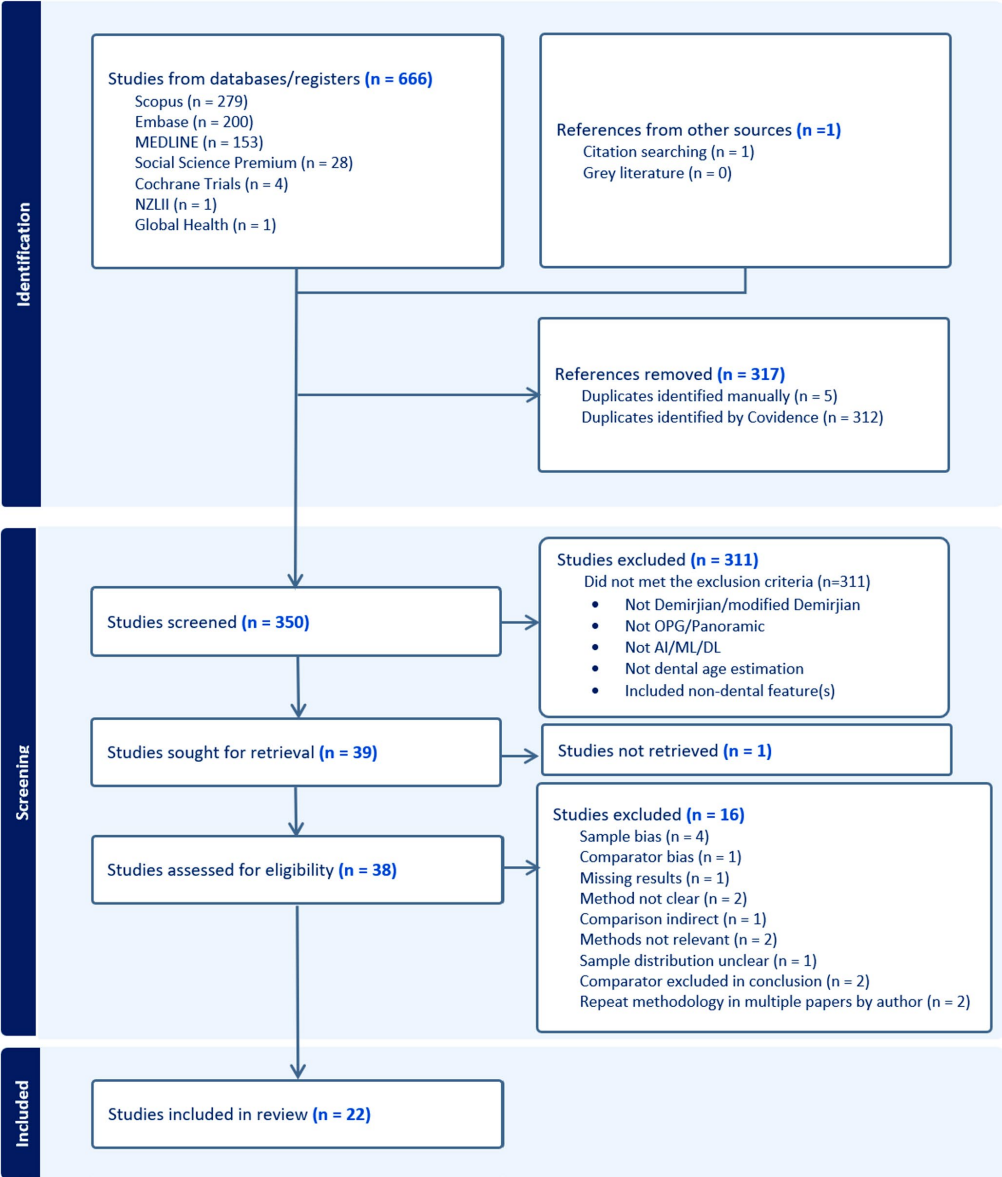
Prisma flow diagram generated by Covidence for the scoping review comparing the manual application of the Demirjian method and artificial intelligence modalities

The first search yielded 595 articles of which 265 were duplicates. From the yield of 330, 20 full text articles were sourced. A yield of 71 came from the second search, of which 30 were duplicates. The yield of 41 articles came from the second search, of which two full-text articles were sourced, one of which was a correction for an article sourced in the first search. A final total of 22 articles from an initial total of 666 articles was used in this scoping review (Fig. 1). The corrected article was combined with the original article as a count of one.

### Characteristics of sources evidence

The included articles were subdivided into three groups according to the following characteristics i.e., Stage Allocation Alone, Stage Allocation Prior to AI; and Demirjian Prior to AI. The groups were tabulated using the headings outlined under Data items (see Tables 1, 2 and 3).

**Table 1:** Demirjian stage allocation (Stage alone Studies)

Authors	Year	*n*	Population	Sex	Sample spread	Tooth features	DAE method: # of Stages	AIgorithm architecture	Reported Metrics	Classification Performance
De Tobel et al.. [5]	2017	400	Belgium	M&F	20 per stage	38	De Tobel: 10	AlexNet 5−fold cross−validation	Mean Rank−N RR, MAD, LWK, ICC, cross−tabulation	MA: 0.51 (mean Rank−I RR) MAD: 0.6 stages LWK: 0.82
Merdietio Boedi et al.. [7]	2020	400	Belgium	M&F	20 per stage	38	De Tobel: 10	DenseNet201 5−fold cross−validation	Accuracy, MAD, Cohen’s Klin, cross−tabulation	*Fully segmented* MA: 0.61 MAD: 0.53 stages Cohen’s Klin: 84%
Banar et al.. [6]	2020	400	Belgium	M&F	20 per stage	38	De Tobel: 10	YOLO−like CNN, DenseNet201, U−Net−like CNN 5−fold cross−validation	MAE (pix); MED (pix); accuracy, MAE (stages), precision, recall, dice, LWK, cross−tabulation, confusion matrix	MA − auto/manual = 54/60 (%) MAE (stages) auto/manual = 0.69/0.51 stages LWK− auto/manual = 79%/84%
Matthijs et al.. [44]	2023	1639	Belgium	M&F	20 per stage	31, 33, 34, 37, 38	De Tobel: 10	DenseNet 5−fold cross−validation	Confusion matrix, mean rank−N, accuracy, MAD, LWK, ICC	Accuracy: 0.53 MAD: 0.71 LWCK: 0.71 ICC: 0.89
Upalananda et al.. [28]	2023	4000	Thai	M&F	15–23yrs	38, 48	Demirjian stages D−H	GoogLeNet Training epoch = 10 Mini batch size = 32	Mean, SD, min, max, cross−tabulation, sensitivity, specificity, PPV, NPV, accuracy	Accuracy: 82.50% (stage range: 87.5–97.5%)
Milani et al.. [67]	2024	3422	USA	M&F	Per stage	38, 48	Modified Demirjian: 10	Custom CNN 5−fold cross validation	Mean, SD, 95% CI, confusion matrix, accuracy	Accuracy: 83.76%
Ong et al.. [68]	2024	5133	S.Korea	M&F	4–16yrs	31–37	Demirjian: 8	YOLOv5, U−Net, EfficientNet Batch size 10 Epochs = 1000	mAP, recall, precision, F1−score, cross−tabulation	MA: 0.995 F1−scores per tooth type I:69.23, C:80.67, PM:84.97, M:90.81

Mean Rank-N *RR* Rank-N recognition rate, *MAD * mean absolute difference, *LWK * linearly weighted kappa coefficient, *ICC* intercorrelation coefficient, *MA* mean accuracy, *MAE* mean absolute error, *MED* mean Euclidean distance, *SD* standard deviation, *min *minimum, *max* maximum, *PPV * positive prediction value, *NPV* negative prediction value,* mAP* mean average precision, *I* incisor, *C * canine, *PM* premolar, *M* molar

**Table 2:** Comparison of studies using Demirjian stages and AI DAE (Stage allocation prior to AI)

Authors	Year	n	Population	Sex	Sample spread	Tooth features	DAE method: # of Stages	AIgorithm architecture	Reported Metrics	Stage Allocation Performance	DAE Performance
Mohammad et al.. [71]	2021	657	Malay	M&F	5–14.99yrs	34 (P1), 35 (P2)	Modified Demirjian C−F, added stages D1−D5	AlexNet Training epochs = 10 Minibatch size = 32	ME, p−value, cross−tabulation, precision, recall, dice (F−score), Jaccard similarity, confusion matrix	MA: 92.5% F1−scores: P1=0.94; P2=0.91	ME Female: P1=−0.23; P2=0.03 ME Male: P1=0.18; P2=−0.02
Han et al.. [9]	2022	10257	Chinese	M&F	5–24yrs	31–38	Demirjian: 8	Chinese MLR formula; ResNet ADSE batch size = 1 ADAE batch size = 32	mean, SD, 95% CI, p−value, MAE, confusion matrix, accuracy (%), LWK	Accuracy: 87.07% MAE: 0.17 stages LWK: 0.80	MAE (man): 1.67yrs MAE (semi): 1.63yrs MAE (auto): 1.03yrs ^a^
Pintana et al.. [27]	2022	1000	Thailand	M&F	15–23yrs; 200/stage	38	Demirjian D−H	ACF detector Resnet50 Mini batch size = 32	Accuracy, sensitivity, specificity, PPV, NPV, confusion matrix	Accuracy (locator)=99.5% Accuracy (classification)=93.66% MSens (classification)=83.25%	Accuracy per age margin ^b^ ± 0.5 yrs = 10.05% ± 1 yr = 26.63% ± 2 yrs = 58.29% ± 4 yrs= 90%+ Overall MAE=1.94yrs
Kumagi et al. [32]	2023	2657	Korean Japanese	M&F	15–23yrs	M1 & M2	Demirjian: 8	DAE: SLP & MLP data mining (DM) Classification: Multivariable LR (Lr and LogR)	MAE, RMSE, sensitivity, specificity, PPV, NPV, AUROC	Internal set: conventional better than DM for males; comparable for females AUROC > 0.92 for all External set: DM & conventional comparable for males; DM better than conventional for females ^c^	MAE DM−Internal: M/F = 1.133/1.278 MAE Conventional−Internal: M/F = 1.016/1.077 MAE DM−External: M/F = 1.249/1.803 MAE Conventional−External: M/F = 1.250/1.717
Mohammad et al.. [4]	2024	4892	Malay	M&F	5.1–15.8yrs	34 (P1), 35 (P2)	Modified Demirjian: C−F	DP−AC Approx 20 epochs	Accuracy, Precision, Recall, F1−score, 95%CI, confusion matrix, ME, p−value	Accuracy: 96.63%	ME F: P1=0.07; P2=0.03 ME M: P1= − 0.03; P2= − 0.02
Zeng et al.. [72]	2024	1486	S. China	M&F	8–24.99	M2 & M3 (36 & 37)	Demirjian	Multiple ML models Step−wise regression Ordered logistic regression 10−fold cross−validation	Accuracy, MAE, mean, max, min, SD, M−W U−test, p−value, Wilcoxon test, R, R−sq, Adj R−sq, SE, R2 change, 95%CI, precision, recall, F1−score, confusion matrix, ROC curves, MSE. RMSE	SVM AUC = 0.9896 (> 18-year group) ^d^	M3R+M3L+M2R+M2L+sex: accuracy = 0.6144 SVM classification accuracy = 0.618 VOTE MAE = 0.7207 (overall) RF MAE = 0.4248 (12–14yr group)

*ME* mean error, *MA* mean accuracy, *SD* standard deviation, *MAE* mean absolute error, *LWK* linearly weighted kappa coefficient, *PPV* positive prediction value, *NPV* negative prediction value, *MSens* mean sensitivity, *ADSE* assisted dental stage estimation, *ADAE* automated dental age estimation, *SD* standard deviation, LWK=; *Lr* linear regression, *LogR* logistic regression, *RMSE* root mean square error, *AUROC* area under the region of curve, *max* maximum, *min* minimum, *M* male, *F* female, *M-W* Mann-Whitney, *R-sq* R squared, *Adj R-sq* adjusted r-squared, *SE* standard error, *ROC* region of curve, *MSE* mean standard error, *RMSE* root mean square error, *M3R* right 3rd molar, *M3L* left 3rd molar, *M2R* right 2nd molar, *M2L* left 2nd molar, *RF* Random Forrest

^a^ 2025 correction to ADSE

^b^ Used Duangto et al. regression equations

^c^ Thresholds = < 12, 12–14, 14–16, 16–18, > 18-year groups

^d^ Threshold = 18-years

**Table 3:** Comparison of studies that tested the Demirjian method before AI DAE (Demirjian prior to AI)

Authors	Year	*n*	Population	Sex	Sample spread	Tooth features	DAE method: # of Stages	AIgorithm architecture	Classification threshold	Metrics reported (classification or DAE)	DAE Performance
Tao et al.. [34]	2020	1636	Chinese	M&F	11–19yrs	31–37	Demirjian: 8 Willems: 8	ML – Multilayer Perceptron Neural Networks Leave-one-out cross-validation		RMSE, MSE, MAE	*MAE: M/F* Demirjian: 1.307/1.364 Willems: 1.291/1.407 MLP: 0.990/1.261
Galibourg et al.. [24]	2021	2230	S. France	M&F	^a^2.18 < 16yrs	31–37	Demirjian: 8 Willems: 8	ML – various	U16	ME, MAE, MSE, RMSE, R2, SD. Bland-Altman graph	MAE-Demirjian: 1.108yr (0.045) MAE-Willems: 0.928yr (0.037) MAE-ML: SVM 0.729(0.025) to BRR 0.812(0.028)
		1340	S. France	M&F	16<24yrs	4x third molars	Hofmann modified Dem: 8	ML – various 10-fold cross-validation	U24		MAE-Hofmann = 1.639 MAE-ML = 1.689 (SVM) to 2.828(BRR)
		3570	S. France	M&F	^a^2.18 < 24yrs	31–37, 4x third molars	Demirjian: 8 Willems: 8 Hofmann modified Dem: 8	ML – various 10-fold cross-validation	U24		MAE: 1.197(SVM) to 2.206(BRR)
Guo et al.. [18]	2021	10257	Chinese	M&F	5–25yr	31–38; 38	Demirjian: 8	SE-ResNet & EfficientNet	14 yr, 16 yr, 18 yr	Accuracy, sensitivity, specificity,	*Age Thresholds (years):14yr 16 yr 18yr* *Teeth 31–38* % Accuracy (manual): 92.5 91.3 91.8 % Accuracy (auto): 95.9 95.4 92.3 *Tooth 38* % Accuracy (manual): 90.0 91.5 91.7 % Accuracy (auto): 93.4 95.7 93.3
Bunyarit et al.. [21]	2022	1015	Malaysian Indian	M&F	5<18yrs	31–38	Chaillet and Demirjian: 8 (8-teeth method only)	ANN- MLP		mean, SD, MD, 95%CI, t, p-value, r	MD-Chaillet & Demirjian M/F: −1.68/−2.56 MD-ANN M/F: 0.03/0.07
Rocha et al.. [78]	2022	1000	SE Brazil	M&F	6–15.99yrs	31–38	Willems: 8	MLR ANN-MLP		mean, SD, ME, MAE, RMSE, Bland-Altman graphs	ME Willems (F/M) = 0.27/0.28 ME MLR(F/M) = 0.54/0.35 ME ANN (F/M) = 0.26/0.24 *Excluding 15yo*: MLR=0.47/0.25 ANN=0.14/0.16 Willems=0.22/0.26
Shan et al.. [31]	2022	1477	S. China	M&F	2<18yrs	31–37	Demirjian: 8 Willems: 8 Modified Willems: 8	Regression Variable leaf node depths, weights & iterations	<16 & <18yrs thresholds:	AD, SD, MAE, p-value, MSE, RMSE, R2	MAE Demirjian: F (<18:<16)/M (<18:<16) = 0.95:0.94/0.96:0.96 MAE Willems: F (<18:<16)/M (<18:<16) = 0.78:0.73/0.77:0.76 MAE Modified Willems: F (<18:<16)/M (<18:<16) = 0.77:0.71/0.76:0.72
Wu et al. (a) [33]	2022	2052	Taiwanese	M&F	2.8<18yrs	31–37	Demirjian 8 Willems 8 ^b^ H-method: 8	ML – various Repeat training – 20 iterations		RMSE, R-sq, MSE, MAE, ME, Bland-Altman graphs, means, SD (ML only)	*MAE ± SD (not reported for non-ML)* GPR = 0.6916 ± 0.001 Han:1.239 Demirjian:1.850 Willems:4.314
Wu et al. (b) [79]	2022	2431	Taiwanese	M&F	3–18yrs	31–37	Demirjian 8 Willems 8	ML – GPR CNN – EfficientNet 5-fold cross-validation		Mean, SD, SEM, p-value	*Estimated age (CA): mean (SD) F/M* CA-Demirjian: −0.818 (0.852)/−0.926 (1.01) CA-Willems: −0.279 (0.792)/−0.468 (0.917) CA-ML: 0.039 (0.736)/−0.050 (0.770) CA-CNN: 0.014 (0.718)/0.007 (0.637)
Yavuz et al.. [80]	2025	1169	Turkish	M&F	5–16yrs	31–37	Willems: 8	CNN – ResNet 5-fold cross validation		mean, SD, ICC, p-value, r, 95% CI, AIC, BIC, RMSE, R-sq, MD, MAE	*MAE* Willems F/M: :0.2 to 1.3yrs/0.06 to 1 yr DL F/M: −2.45 to 1.38yrs/−1.95 to 1.56yrs *RMSE/R-square* Willems: 0.939/0.897 Deep Learning: 1.670/0.676

*RMSE* root mean square error, *MSE* mean square error,* MAE* mean absolute error, *U16* under 16-years, *U24* under 24-years, *ME* mean error, *r* correlation coefficient, *R-sq* r-squared, *SD* standard deviation, *MD* mean difference, *MLR* multilinear regression, *AD* average difference, *SEM* standard error of mean, *GPR *Gaussian process regression, *ICC* intraclass correlation coefficient, *AIC* Akaike information criterion, *BIC* Bayesian-Schwarz criterion

^a^ results given in the study are from 4 years not 2.18yrs

^b^ H-method = Han modified Demirjian – Pan et al. age range 5−16.99 year-olds

### Critical appraisal within sources of evidence

Data extracted from each source of evidence was tabulated according to group allocation to assist comparisons (Tables 1, 2 and 3). Tabulations included numerical data such as sample size, sample age range, number of stages used, and reported metric results.

### Results of individual sources of evidence

A summary of the appraisal of each source of evidence is included under one of the three following allocated groups.

#### Stage allocation alone

This group included seven stage allocation studies (Stage Allocation Alone) that focused on AI methods to automate Demirjian staging. Table 1 summarises the data items evaluated in this review.

De Tobel et al. [5] developed a modified Demirjian staging index (Online Resource 3. Table 1.). The study used an automated approach but still required bounding boxes to fix the region of interest. Percentage accuracy of stage allocation for tooth 38 using Rank-N recognition rate was reported and found to be similar between human observers in the study and the automated technique. Accuracy was defined as the first rank recognition rate i.e., Rank-I RR. The linearly weighted kappa (LWK) coefficient indicated the most misclassified stages were neighbouring stages only. The authors warned that errors in the supervised annotation of the training data will be copied by the automated technique.

Merdietio Boedi et al. [7] attempted to improve the automated 10 stage allocation study by De Tobel et al. They removed surrounding structures (e.g., periodontal ligament, bone, mandibular canal) to delineate the third molar, comparing three types of segmentations i.e., bounding box (BB), rough segmentation (RS), and full segmentation (FS). They also replaced the AlexNet architecture with DenseNet201. FS gave the best results, outperforming BB by 7%. Misclassified stages were neighbouring stages only. Reported staging performance was better than De Tobel and reached similar performance to humans.

Banar et al. [6] replicated the method of De Tobel et al. [5] with the same sample but utilised a three-step method to classify tooth 38. Step one involved a YOLO-like CNN/DenseNet201 architecture to apply bounding boxes and feature mapping; step two utilised a U-Net like CNN for segmentation; and step three used a U-Net like CNN to automate tooth staging. They were able to achieve a slightly better mean accuracy for stage allocation, but were unable to achieve better than Merdietio Boedi et al. [7]. They found “discerning adjacent stages (especially stages 7–9) remained challenging for the automated DL approach”.

An automated staging technique developed by Matthijs et al. [44] aimed at 400 radiographs per tooth type and 20 per stage per sex, however this was not achieved for lower stages for most tooth types. De Tobel’s 10 stage method was utilised by the human experts. The relevant teeth were placed in bounding boxes and images resized. A CNN model was used to establish an integrated automated staging technique for the different mandibular tooth types. The authors suggest that the developmental status of mandibular molars can be considered in an automated method for DAE, however incisors may hinder DAE.

The semi-automatic stage assessment study by Upalananda et al. [28] assigned dentists to manually locate, crop, and rotate bilateral lower third molar images. Exclusion criteria were not stated. Images from the same individual were excluded before descriptive statistical analysis, removing some potential bias. The sample was imbalanced across age groups and between sexes but reasonably distributed across stages. The study presented cross tabulations between the observer and the pre-trained GoogLeNet model. All misclassifications from the model deviated by one stage from the developmental stage assessed by the observer. “The method’s performance was comparable with human assessment.” The study compared its results to studies using a modified Demirjian method [5, 6] and achieved a ‘acceptable’ mean accuracy comparable to other studies using the manual method [69].

Milani et al. [67] introduced a ML model to allocate modified Demirjian stages (Online Resource 3. Table 2) of bilateral third molars. No rationale was given to the modification of the Demirjian stages and for using absolute measures in stage 5. Manual cropping, resizing, and classification were undertaken by two clinical scientists. The method employed a DNN model for region of interest extraction, pre-filtering, and extensive data augmentation (weighted data sampling, auto contrast, translation) to expand the sample by merging trainable filters with elements of a pre-trained network (transfer learning). Focal loss was incorporated to address class imbalances and prioritize learning from difficult examples. The EfficientNet method achieved the highest classification accuracy of 83.7%, with cross-tabulations being very similar to the ground truth. Misclassifications were greater than 1-stage across the sample. The sample was imbalanced across stages and sex.

Ong et al. [70] classified four tooth types utilising a three-step automated procedure i.e., detection, segmentation, and classification. YOLOv5, U-Net, and EfficientNet models were trained and used for each Demirjian stage. Mean average precision (mAP) was 0.995 for detection and 0.978 for segmentation. It was stated that use of the proposed model would eliminate disagreements between observers, however the cross-tabulation results are comparable between the expert and AI model. The authors recommended multiple tooth assessments over single tooth types to reduce the risk of broad age ranges. The authors noted limited data for early stages likely introduced bias, and that manual intervention is still necessary to minimise errors from DL models, while DL can reduce observer fatigue and bias. The authors stated the proposed model served as a supportive tool for dentists.

#### Stage allocation prior to AI

Table 2 summarises the data items evaluated in the studies which compared stage allocations between humans and AI models prior to training and developing AI models to allocate stage and then predict dental age.

Mohammad et al. [71] reclassified Demirjian’s staging for their assessment of the mandibular premolars (P1 and P2) after applying the traditional Demirjian method to the sample, which resulted in underestimation of males and overestimation of females. Stages below C were not considered. The sample distribution was reported as unbalanced with limited data for stages A and B. Based on accuracy obtained from the classification performance, new stages were formulated (Online Resource 3. Table 3). Automated staging performance was comparable to expert staging. The study reported a high variance in the sample age which was reflected in the significant variation in mean error. The DCNN produced a classification accuracy of 92.5% however, the authors then stated that, due to the imbalance, classification accuracy is an inappropriate measurement and report precision and recall metrics as an alternative, reporting F-scores for the DCNN model alone. The study concludes that the semi-automatic system was superior to the traditional method. DAE performance was compared to the results of two systematic reviews with meta-analyses assessing the Willems method (a modified Demirjian method) [73–75].

Han et al. [9, 76] tested the performance of three models i.e., manual DAE (MDAE), automated stage evaluation (ADSE), and end-to-end automated DAE (ADAE), to compare a stepped approach from human application of a Chinese regression formula to end-to-end automation. The stage imbalanced dataset resulted in only stages D, E, and H yielding accuracy of > 70%. The consistency between the experts (Linear Weighted Kappa (LWK) = inter:0.874/intra:0.967) and the ADSE model (LWK = 0.801) indicated the model nearly approached the performance of the expert inter-observer. The study compared classification-stage performance to studies using only third molars, most teeth had reached stage H, and the DAE performance did not significantly improve on manual method. The authors advised the automated classification model needs to improve for more accurate classification of stages. The ADAE model was found to outperform the other two models by approximately 1/3 (2025 republished version) [76]. Grad-CAM indicated other features were assessed, including maxillary sinus and the space distal to the third molar. Although sex-matched MLR regression formulas for the ADSE models were utilised, no sex-specific results were reported in the study [9].

Pintana et al. [27] employed a three-step technique comparing classification results to the experts-evaluated ground truth; reporting sensitivity, specificity, PPV, NPV, and accuracy. An ACF detector automated tooth location in step one; a pre-trained ResNet50 architecture categorised stages; and finally dental age predictions were calculated using the Duangto et al. Equation [77]. The study was unable to compare the method to other recent studies due to differences in datasets, experimental settings, and evaluation metrics. The authors indicated that hardware restrictions may have affected performance. For DAE, Duangto et al. sex-matched regression equations (formulated from a Thai sample using left and then right third molars) were applied and percentage accuracy reported for 0.5-year intervals. Sex-matched results were not reported. Accuracy was low for acceptable age-ranges of ≤ ± 2years. Stage allocation was comparable to the experts. Target and reference populations matched.

In a data mining (DM) study by Kumagai et al. [32] a Korean sample was used to train and test (internal) models, while a Japanese sample was used in the external test. The study compared the accuracy of DM models with conventional regression models. The second and third molars were staged according to Demirjian’s method. Multivariable linear and logistic regression were then performed to develop new population-specific regression formulae to estimate age for comparison to DM models. The conventional models performed slightly better than the DM models for sex-matched DAE. Conventional classification performed best for Korean males. Of the DM models, logistic regression was found to be optimal for classification for the sex-matched Korean sample. Overall, the accuracy of DAE and classification performance of the Japanese sample was less accurate compared to the Korean sample, indicating the limitation of multi-population application.

In 2024, Mohammad et al. [4] followed their 2021 study [71] examining the accuracy of classifying P1 and P2 teeth, utilising their proposed DCNN model. The model was a four-step method, including preprocessing by one expert (data labelling and annotation). According to the study the DCNN model improved accuracy; but there was no difference between the 2021 and 2024 male results for CA and PA for P1 (ME and 95%CI did differ between the two studies for male P1). The confusion matrix indicated similar results between true and predicted classifications. Sex-matched results were reported; age was underestimated for females and overestimated for males.

Zeng et al. [72] employed a two-step approach to combine classification and prediction models. Two experts scored and allocated Demirjian stages to bilateral second (M2) and third (M3) molars. Sex-matched descriptive statistics were reported for each developmental stage (A-H). The sample was categorised into five age groups: <12, 12–14, 14–16, 16–18, > 18 years with the classification model, followed by application of a prediction model within each age group. Only a 1.5% improvement was found using the ML multiclass classification model, compared to the LR model. When converted to a binary classification problem a pronounced advantage resulted. A joint model for bilateral M2 and M3 was developed and found to be more efficient than M3 alone for DAE. Age prediction accuracy ranged from MAE = 0.5081 (12-14years) to MAE = 1.6290 (≥ 18years) The bilateral M3 + M2 + sex model gave the highest accuracy. The DAE model is yet to be validated for other population groups.

#### Demirjian prior to AI

A total of nine studies were allocated to the Demirjian Prior to AI group for analysis. Data evaluated was tabulated in Table 3.

The 2020 study by Tao et al. [34] employed a Multi-layer Perceptron (ML-P) model to predict dental age for their sample. They compared the predictions to those of experts using the traditional Demirjian and Willem’s (modified Demirjian) methods. Leave-one-out cross-validation was used to avoid over-fitting during training stage. Once trained using the Demirjian stages, the model was used to predict age. An experiment was then conducted using Demirjian and Willem’s converted features to train the model. The experiment showed improved accuracy from the first ML-P model and particularly for males compared to females.

The Galibourg et al. [24] study can be divided into three parts. For the U16-year group, teeth 31–37 were scored and compared to CA. The ME was then compared to those from the ML regression methods. Study results were reported for 4–16-years and/or 4–24-years where the ML methods were superior to traditional methods at DAE. For the U24-year group, it was reported that ML algorithms were better than the traditional methods however, this is an overall assumption, since the Hofmann method [81] employed was better for assessing the third molars (lowest ME, MAE, MSE, and RMSE). The Hofmann method was used since Demirjian and Willems methods cannot be applied to over 16-year-olds. The study showed the greatest error from 17 to 20 years, which may be explained by lower sample numbers in these groups. The study acknowledged the limitation of applying the 1973 French-Canadian reference standards to different groups. The Hofmann method was based on a German sample and the Galibourg et al. sample was from Southern France. Sex-matched data was only presented in the Bland-Altman plots.

Guo et al. [18] set age thresholds of 14, 16, and 18 years old, comparing the manual technique of Demirjian and automatic techniques using SE-ResNet (one model) and EfficientNet (three models). All methods produced accuracy of over 90%. The accuracy, sensitivity, and specificity for teeth 31–38 were mostly higher than for tooth 38 for the 14 and 16-year thresholds, with the reverse being shown for tooth 38 for the 18-year threshold. Similar results between machine and manual methods were obtained in the study. Specificity and sensitivity of the manual method were highest for the 18-year threshold. The authors suggested the stability of the manual method may be related to the ability of humans to avoid the feature interaction problem and noise interference, due to professional knowledge and experience interpreting imaging.

Bunyarit et al. [21] manually applied the Chaillet and Demirjian method [82] to calculate DAE. ANN, employing MLP, produced new modified scores to calculate new dental age (NDA). While the Chaillet and Demirjian method underestimated DA, the ANN adapted scores improved DAE by narrowing the gap between CA and DA to 12-days (boys) and 25-days (girls). Reported mean differences for ANN treated samples were all under 0.23-years, but SDs were higher than 1-year for both sexes (males 11 < 14-years; females 11 < 17-years). The modified scores are yet to be validated.

Rocha et al. [78] employed the Willems method [75] for DAE, with sex-matched results reported. Comparison between the traditional method, MLR and ANN models revealed that the ANN model performed better than the MLR model. The Willem’s method remained optimal for most age intervals for females and > 12-year intervals for males. All models were inaccurate for the 15–15.99.99-year group.

In the study by Shan et al. [31] dental ages were calculated by applying the Demirjian [59] and the Willems [75] methods and compared to CA. The Willems method gave the lowest error and thus utilised to generate a new model, producing new weighted scores. Age estimates were improved minimally compared to boosting ML algorithms trained with the Demirjian stages for sex-matched data. GBDT achieved MAEs below 0.25 for all age groups in the test set. The study used an imbalanced sample across sex-matched age groups. The authors did not consider this problematic nor affected results, although the sample was reported as a limiting factor. The results have not been validated yet.

Wu et al. (a) [33] trained six ML DAE methods by inputting allocated Demirjian stages of teeth 31–37 and CA in the training dataset. Model performances were compared to the H-method [83], the Demirjian [59], and the Willems [75] methods. The H-method refers to the sex-matched maturity score conversion tables developed by Pan et al. (2021), derived from the Demirjian method. The ML-assisted models reduced MAE to less than one-year overall for the combined-sex sample. The Gaussian process regression performed the best out of the ML models and the H-method was best of the non-ML models.

Wu et al. (b) [79] assessed the images of a group of healthy children and a group of children with delayed growth (GD) using traditional and AI-assisted standards. The traditional methods included the Demirjian [59] and Willems methods [75], plus a modified Taiwanese developed, specific version of Demirjian. Both staging (TDS) and image features were separately used as input parameters, plus sex. Inputs from healthy children were used for training the ML and CNN models to develop population specific standards, and then compared their efficacy to traditional methods, testing the validation and the growth delayed sets. The CNN model was most accurate for the healthy, while the Willem’s method was most accurate for the GD children. The ML TDS + sex model had a higher age prediction accuracy then the traditional models with the same parameters. The study sample was aged 3–18-years, however the Demirjian method is only applicable to children aged 3−16-years and the H-method is based on a sample aged 5–16.99.99-years; the study does not indicate if the mature individuals were excluded.

Dental ages were evaluated by two experts using the Willems [75], Cameriere-Europe [84], and London Atlas [85] methods by Yavuz et al. [80]. Images were downsized before imputation. A ResNet transfer learning pre-trained model was employed for DL. MAE was reported for each sex-matched age group for the three traditional methods and the ML model. The DL algorithm did not produce good results without ‘hand-crafted features’ requiring expert knowledge. The DL models gave similar results to traditional methods, but the DL method was the least accurate, with the lowest determination coefficient. No method emerged in this study as suitable for predicting age in the Turkish population. The authors recommended larger datasets to achieve increased accuracy i.e., > 1000 per group for 98% accuracy and > 4000 per group for 99% accuracy.

### Synthesis of results

Within the *Staging Alone* group, two articles used the original Demirjian stages [26, 68], while the others modified stages. Only three studies were directly comparable, using the same sample distribution, tooth feature, and staging method [5–7]. Sex-matching was irrelevant and not included. Five studies reported similar results to the conventional methods [5–7, 28, 44].

Aside from the two Mohammad et al. methods, all studies within the *Staging Prior to AI* group assessed different tooth features and applied unmodified Demirjian stages. Three studies did not provide sex-matched results [18, 24, 33].

Five studies within the *Demirjian Prior to AI* group tested the traditional and a modified version(s) of the Demirjian method on specific population groups prior to training AI models to automate DA predictions [24, 31, 33, 34, 79]; three tested a modified version of the Demirjian method alone [21, 78, 80]; one tested the Demirjian method solely [18]. Six tabulated sex-matched results [21, 31, 34, 78–80]. Two studies reported combined sex results [24, 33].

Comparison between the three groups was difficult due to different methodologies/statistical approaches between groups e.g., the stage alone group did not perform DAE and comparison with experts was solely based on staging accuracy; the Staging Prior to AI group did not perform DAE using the traditional method on their population sample prior to calculating DAE from new regression formulae and AI models developed, therefore comparisons to experts were related to staging alone and not necessarily to the traditional method per se. Six of the nine studies within the third group were able to make direct comparisons to the application of the traditional Demirjian method [18, 24, 31, 33, 34, 79], while three used modified Demirjian methods (two used Willems [78, 80]; one used Chaillet and Demirjian [21]).

Of the 22 studies included in this review, nine showed similar performance between experts manually applying Demirjian stages with/out scoring and AI models [4–7, 18, 27, 28, 31, 44]; four reported the AI models were better [9, 21, 24, 67]; two reported the AI models were worse [32, 78], four reported mixed results [4, 33, 68, 79], one reported that neither were applicable to their study population [80], and two did not report comparison between experts and AI [34, 72].

## Discussion

There is a rapidly increasing trend in the use of AI to improve accuracy and efficiency in DAE, yet many studies fail to address issues raised from research undertaken using traditional methods [86]. Several studies were excluded from this review for violating more than one such issue. Other excluded studies contained samples with age ranges that included teeth in regressive phases that can increase error rates significantly; reporting of mean errors for these samples does not assist DAE of subadults and should be analysed separately or separate results reported for developing and mature teeth. Furthermore, arranging samples into more than 1-year groups and/or presenting overall means or point estimates, does not assist age estimation of the living where thresholding is utilised when an age estimation of an individual comes into question, from a legal or human rights perspective. Although AI has demonstrated greater efficiency in some areas of DAE, it cannot replace the experience of a dentist, particularly when analysing complex radiographic morphologies [22]. While some studies incorporate limited statements of experience of dental ‘experts’, others do not. Furthermore, the definition of who qualifies as an expert has not been clearly defined in this field.

The 2009 National Academy of Sciences report states that “since researchers are, by definition, creating new understanding, they must be as cautious as possible before asserting a new ‘truth’. Also, because researchers are working at a frontier, few others may have the knowledge to catch and correct any errors they make” [87]. The race to be the first to develop new methods should not mean lessons from previous relevant bodies of research can be ignored [86].

In this scoping review the comparison between the studies was challenging, due to the wide variation in different tooth types, sample size and distribution, different statistical methods applied, results reported, and the presence of sample bias. Only three studies in this review were comparable in terms of sample size, distribution, dental features assessed, population, and DAE method used. They varied in their AI architecture approach upon discovering possible areas for improvement [5–7].

It was anticipated that AI would outperform experts in stage allocation (Stage Allocation Alone). The lower stage allocation accuracy of some studies is possibly a reflection of the difficulty in assessing third molars for both machines and experts. Sample size and the use of modified Demirjian stages may also have affected outcomes. Those with larger samples and/or used unmodified Demirjian stages had higher accuracy.

Studies that trained models with Demirjian stages (Stage Allocation Prior to AI) were able to produce good classification accuracy through employing locator algorithms that utilised more features than delineated teeth alone. DAE performance was not strong in this group, and results may have been skewed for those that did not report sex matched results [18, 24, 33].

For the studies that compared direct application of the Demirjian or modified Demirjian methods to a proposed AI method (Demirjian Prior to AI), the outcome was as predicted i.e., the proposed study methods were likely to outperform the traditional method where the populations differed from the Demirjian French-Canadian sample [21, 24, 31, 78]. In this group, bias may have been imposed by reporting of combined sex results [24, 33]. Combining sexes can hide relevant information [34, 88]. If an assumption was made based on combined results that a method was good for both sexes, the method could produce incorrect results with real-life consequences.

Bias may also have been introduced using age ranges that differed between target and reference samples [33, 34, 89]. It is unclear in these studies if dentally mature individuals over 16-years old were eliminated from statistical analysis. Once the apices of the seven lower teeth (I1 to M2) have closed we cannot tell from the teeth alone if a person is 16-years old (early 20 s if the third molar is included) or 80-years old, for example, therefore, overall error rates will be unhelpful for immature individuals.

Consideration of tooth selection for analysis is relevant as it is known that third molars are notorious for variation and the use of 2-D imaging (OPG) limits viewing and potentially excludes otherwise usable data. Furthermore, DAE comparisons with traditional methods, which utilise seven or eight teeth, are difficult if studies select different combinations of teeth to analyse [4, 27, 72].

While there is little consensus on what is reliable or valid when considering DAE accuracy, Ritz-Timme et al. (2000) consider reported errors of 0.5–1.5 year up to the age of 14-years, with 2 years up to the age of 18-years, as acceptable. Liversidge et al. (2010) and Tangmose et al. (2015) report standard deviations of approximately 0.7 to 1-year up to 8–9 years of age, with 18 months to 2-years for third molar stages, as acceptable. “Accuracy will be influenced by age range and sample structure” and whether “a child is maturing at an average rate” (Liversidge et al., 2010, Tangmose et al., 2015) [54, 90]. Although some of these studies report AI outperforming traditional methods, their accuracy may not satisfy real-world application requirements, and no consensus was found in this review. Accuracy, or validity is defined as “the degree of conformity of a measured or calculated quantity to its actual true value” [91]. Accuracy has been defined by the studies assessed in this review as either mean error or mean difference; both terms were used to demonstrate the difference between CA and DA.

Multiple authors report the time inefficiency of manual application of dental age estimation techniques [12, 92] while others have found the application of traditional methods to be fast, easy, and inexpensive [93]. In the real-world, 10 min per patient (as report by Kapoor and Jain 2018 when applying the polynomial equations and tables of Demirjian and Chaillet 2004 [82, 92]) is not particularly burdensome and manual application of techniques is arguably cheaper than developing validated automated methods [7, 8, 25, 71]. Some authors have argued that mass fatality events involving large numbers of victims would require automated methods to hasten the identification process [4]. There are several limiting factors to this argument e.g., validated population-specific reference data availability [41, 94, 95], available evidence to assess and recoverability, regional economic and political factors.

As observed with traditional AE methods there may also be a risk of bias introduced by the type of regression analysis AI models use. The studies in this review largely did not describe in detail the methods underlying the AI models. While the limitations of manual methods have been well documented, the trustworthiness and integrity of AI systems should also be investigated for application in forensic fields. Experts will be called upon to give evidence in court where they may very well be asked to detail all the processes that transform data into reliable results. Some methods behave like black boxes (MLP, RF, SVM), while others are perfectly explainable (polynomial regression, decision tree). The methods the least easy to interpret give the best results on all the metrics e.g., SVM, RF, MLP [24]. Galibourg et al., Kumagai et al., Rocha et al., Wu(a) et al., and Wu(b) et al. [24, 32, 33, 78, 79], utilised a mix of explicable and less explicable regression methods, while all others used less explicable methods or did not describe the regression method used. Deep learning-based DAE methods make it almost impossible to present the scientific basis of age estimation, such as the age indicators used in the estimation. Commonly, algorithm developers cannot adequately explain how results are arrived at. In addition, there has been legal controversy around who/what performed examinations. Therefore, deep learning-based age estimation is difficult to accept by administrative and judicial agencies [32].

## Conclusion

This scoping review has shown that reliable, applicable, and efficient AI methods for DAE, regardless of how the Demirjian method was utilised, are not yet available to replace current methods. This technology is progressing rapidly, but it is often challenging to directly compare studies. Sample description, size, and distribution continue to be the main areas likely to be introducing bias. Statistical analysis varied immensely, as did the selection of metrics and reporting of results. Greater consistency of these factors is necessary to compare studies.

Utilising Demirjian stages to assist training of AI models can improve accuracy, however human oversight is still required, and new methods need validation. DAE research should be undertaken by experts in the field and with the end-user in sight i.e., forensic and general practitioners. More research is needed to better understand how AI models undertake predictions to make them suitable for real-world use, as error in age estimation potentially has severely negative impacts. The exclusion of meaningful information, results, and clarification by authors in many studies in this review has further hindered the application of these techniques into the forensic and legal domain. Due to the complexity of AI systems, experts need to be able to visualise, interpret, and explain, in easily understood terms, how the models work to be accepted by legal systems requiring DAE in legal proceedings. Methods will be questioned by the triers of fact when human rights are at stake. Proposed methods also need to be easily applicable, reliable, population relevant, and reproducible for use in general dental clinical practice.

## Supplementary Information

Below is the link to the electronic supplementary material.

Supplementary Material 1 (PDF 175 KB)

Supplementary Material 2 (PDF 209 KB)

Supplementary Material 3 (PDF 144 KB)

## Data Availability

Data

## Abbreviations

SB: Dr. Stephanie Baylis
AF: Dr. Adrian Fernandez
JD: Dr. Joanna Francis Dipnall
RB: Professor Richard Bassed

## References

[1] Adserias-Garriga J (2019) Chapter 6 - evolution of methods and state-of-the-art in dental age estimation. In: Adserias-Garriga J (ed) Age estimation. Academic Press, pp 77–87

[2] Schmeling A, Geserick G, Reisinger W, Olze A (2007) Age estimation. Forensic Sci Int 165:178–181. 10.1016/j.forsciint.2006.05.016 16782291 10.1016/j.forsciint.2006.05.016

[3] Schmeling A, Grundmann C, Fuhrmann A et al (2008) Criteria for age estimation in living individuals. Int J Leg Med 122:457–460. 10.1007/s00414-008-0254-2 10.1007/s00414-008-0254-2 18548266

[4] Mohammad N, Ahmad R, Gaus MHA, Kurniawan A, Yusof M (2024) Accuracy of automated forensic dental age estimation lab (F-DentEst Lab) on large Malaysian dataset. Forensic Sci Int 361:112150. 10.1016/j.forsciint.2024.112150 39047517 10.1016/j.forsciint.2024.112150

[5] De Tobel J, Radesh P, Vandermeulen D, Thevissen PW (2017) An automated technique to stage lower third molar development on panoramic radiographs for age estimation: a pilot study. J Forensic Odontostomatol 2:49–60 PMC6100230 29384736

[6] Banar N, Bertels J, Laurent F et al (2020) Towards fully automated third molar development staging in panoramic radiographs. Int J Leg Med 134:1831–1841. 10.1007/s00414-020-02283-3 10.1007/s00414-020-02283-3 32239317

[7] Merdietio Boedi R, Banar N, De Tobel J, Bertels J, Vandermeulen D, Thevissen PW (2020) Effect of lower third molar segmentations on automated tooth development staging using a convolutional neural network. J Forensic Sci 65:481–486. 10.1111/1556-4029.14182 31487052 10.1111/1556-4029.14182

[8] Kahaki SMM, Nordin MJ, Ahmad NS, Arzoky M, Ismail W (2020) Deep convolutional neural network designed for age assessment based on orthopantomography data. Neural Comput Appl 32:9357–9368. 10.1007/s00521-019-04449-6

[9] Han M, Du S, Ge Y et al (2022) With or without human interference for precise age estimation based on machine learning? Int J Legal Med 136:821–831. 10.1007/s00414-022-02796-z 35157129 10.1007/s00414-022-02796-z

[10] Aliyev R, Arslanoglu E, Yasa Y, Oktay AB (2022) Age estimation from pediatric panoramic dental images with CNNs and lightGBM. In: TIPTEKNO 2022 - medical technologies congress, proceedings, pp 1–4. 10.1109/TIPTEKNO56568.2022.9960211

[11] de Back WSS, Wagner S, Marré B, Roeder I, Scherf N (2019) Forensic age estimation with Bayesian convolutional neural networks based on panoramic dental X-ray imaging. In: Proceedings of machine learning research MIDL 2019 conference, pp 1–4

[12] Vila-Blanco N, Carreira MJ, Varas-Quintana P, Balsa-Castro C, Tomas I (2020) Deep neural networks for chronological age estimation from opg images. IEEE Trans Med Imaging 39:2374–2384. 10.1109/TMI.2020.2968765 32012002 10.1109/TMI.2020.2968765

[13] Wallraff S, Vesal S, Syben C, Lutz R, Maier A (2021) Age Estimation on Panoramic Dental X-ray Images using Deep Learning. In: Palm C, Deserno TM, Handels H, Maier A, Maier-Hein K, Tolxdorff T (eds) Bildverarbeitung für die Medizin 2021. Springer Fachmedien Wiesbaden Wiesbaden, pp 186–191

[14] Galante N, Cotroneo R, Furci D, Lodetti G, Casali MB (2023) Applications of artificial intelligence in forensic sciences: Current potential benefits, limitations and perspectives. Int J Legal Med 137:445–458. 10.1007/s00414-022-02928-5 36507961 10.1007/s00414-022-02928-5

[15] Vila-Blanco N, Varas-Quintana P, Tomas I, Carreira MJ (2023) A systematic overview of dental methods for age assessment in living individuals: from traditional to artificial intelligence-based approaches. Int J Legal Med 137:1117–1146. 10.1007/s00414-023-02960-z 37055627 10.1007/s00414-023-02960-z PMC10247592

[16] Mualla NH, Houssein ER, Hassan M (2020) Dental age estimation based on X-ray images. Comput Mater Continua 62:591–605. 10.32604/cmc.2020.08580

[17] Kim S, Lee YH, Noh YK, Park FC, Auh QS (2021) Age-group determination of living individuals using first molar images based on artificial intelligence. Sci Rep 11:1073–1084. 10.1038/s41598-020-80182-8 33441753 10.1038/s41598-020-80182-8 PMC7806774

[18] Guo YC, Han M, Chi Y et al (2021) Accurate age classification using manual method and deep convolutional neural network based on orthopantomogram images. Int J Legal Med 135:1589–1597. 10.1007/s00414-021-02542-x 33661340 10.1007/s00414-021-02542-x

[19] Baydogan MP, Baybars SC, Tuncer SA (2023) Age-Net: an advanced hybrid deep learning model for age estimation using orthopantomograph images. Traitement du Signal 40:1553–1563. 10.18280/ts.400423

[20] Čular LTM, Subašić M, Šarić T, Sajković T, Vodanović M (2017) Dental age estimation from panoramic X-ray images using statistical models. In: Proceedings of the 10th international symposium on image and signal processing and analysis. IEEE Piscataway, NJ, pp 25–30

[21] Bunyarit SS, Nambiar P, Naidu M, Asif MK, Poh RYY (2022) Dental age estimation of Malaysian Indian children and adolescents: applicability of Chaillet and Demirjian's modified method using artificial neural network. Ann Hum Biol 49:192–199. 10.1080/03014460.2022.2105396 35997704 10.1080/03014460.2022.2105396

[22] Nino-Sandoval TC, Doria-Martinez AM, Escobar RAV et al (2024) Efficacy of the methods of age determination using artificial intelligence in panoramic radiographs - a systematic review. Int J Legal Med 138:1459–1496. 10.1007/s00414-024-03162-x 38400923 10.1007/s00414-024-03162-x

[23] Shen S, Yuan X, Wang J, Fan L, Zhao J, Tao J (2022) Evaluation of a machine learning algorithms for predicting the dental age of adolescent based on different preprocessing methods. Front Public Health 10:1–9. 10.3389/fpubh.2022.1068253 10.3389/fpubh.2022.1068253 PMC9751184 36530730

[24] Galibourg A, Cussat-Blanc S, Dumoncel J, Telmon N, Monsarrat P, Maret D (2021) Comparison of different machine learning approaches to predict dental age using Demirjian's staging approach. Int J Legal Med 135:665–675. 10.1007/s00414-020-02489-5 33410925 10.1007/s00414-020-02489-5

[25] Khanagar SB, Albalawi F, Alshehri A et al (2024) Performance of artificial intelligence models designed for automated estimation of age using dento-maxillofacial radiographs-a systematic review. Diagnostics (Basel) 14:1–22. 10.3390/diagnostics14111079 10.3390/diagnostics14111079 PMC11172066 38893606

[26] Milošević D, Vodanović M, Galić I, Subašić M (2022) Automated estimation of chronological age from panoramic dental X-ray images using deep learning. Expert Sys Appl 189:116038. 10.1016/j.eswa.2021.116038

[27] Pintana P, Upalananda W, Saekho S, Yarach U, Wantanajittikul K (2022) Fully automated method for dental age estimation using the ACF detector and deep learning. Egypt J Forensic Sci 12:1–12. 10.1186/s41935-022-00314-1

[28] Upalananda W, Wantanajittikul K, Na Lampang S, Janhom A (2023) Semi-automated technique to assess the developmental stage of mandibular third molars for age estimation. Aust J Forensic Sci 55:23–33. 10.1080/00450618.2021.1882570

[29] Shi Y, Ye Z, Guo J et al (2024) Deep learning methods for fully automated dental age estimation on orthopantomograms. Clin Oral Investig 28:198. 10.1007/s00784-024-05598-2 38448657 10.1007/s00784-024-05598-2

[30] Büyükçakır B, Bertels J, Claes P, Vandermeulen D, de Tobel J, Thevissen PW (2024) OPG-based dental age estimation using a data-technical exploration of deep learning techniques. J Forensic Sci 69:919–931. 10.1111/1556-4029.15473 38291770 10.1111/1556-4029.15473

[31] Shan W, Sun Y, Hu L et al (2022) Boosting algorithm improves the accuracy of juvenile forensic dental age estimation in southern China population. Sci Rep 12:15649. 10.1038/s41598-022-20034-9 36123377 10.1038/s41598-022-20034-9 PMC9485148

[32] Kumagai A, Jeong S, Kim D, Kong HJ, Oh S, Lee SS (2023) Validation of data mining models by comparing with conventional methods for dental age estimation in Korean juveniles and young adults. Sci Rep 13:726. 10.1038/s41598-023-28086-1 36639726 10.1038/s41598-023-28086-1 PMC9839668

[33] Wu TJ, Ling Tsai C, Huang YH, Fan TY, Chen YP (2022) Efficacy of machine learning assisted dental age assessment in local population. Legal Medicine 59. 10.1016/j.legalmed.2022.102148 10.1016/j.legalmed.2022.102148 36223694

[34] Tao J, Wang J, Wang A et al (2020) Dental age estimation: a machine learning perspective. Springer International Publishing AG Switzerland.:722–733

[35] Bunyarit SS, Jayaraman J, Naidu MK, Yuen Ying RP, Nambiar P, Asif MK (2019) Dental age estimation of Malaysian Chinese children and adolescents: Chaillet and Demirjian’s method revisited using artificial multilayer perceptron neural network. Aust J Forensic Sci 52:681–698. 10.1080/00450618.2019.1567810

[36] Bunyarit SS, Nambiar P, Naidu MK, Ying RPY, Asif MK (2021) Dental age estimation of Malay children and adolescents: Chaillet and Demirjian's data improved using artificial multilayer perceptron neural network. Pediatr Dent J 31:176–185. 10.1016/j.pdj.2021.06.002

[37] Koch RM, Mentzel HJ, Heinrich A (2025) Deep learning for forensic age estimation using orthopantomograms in children, adolescents, and young adults. Eur Radiol. 10.1007/s00330-025-11373-y 10.1007/s00330-025-11373-y PMC12165891 39862249

[38] De Tobel J, Phlypo I, Fieuws S, Politis C, Verstraete KL, Thevissen PW (2017) Forensic age estimation based on development of third molars: a staging technique for magnetic resonance imaging. J Forensic Odontostomatol 35:117–140 29384743 PMC6100221

[39] Pham CV, Lee SJ, Kim SY, Lee S, Kim SH, Kim HS (2021) Age estimation based on 3D post-mortem computed tomography images of mandible and femur using convolutional neural networks. PLoS One 16:e0251388. 10.1371/journal.pone.0251388 33979376 10.1371/journal.pone.0251388 PMC8115850

[40] Liversidge HM (2024) Commentary on "new systems for dental maturity based on seven and four teeth" Demirjian and Goldstein, Annals of Human Biology, 1976,3,411-421. Ann Hum Biol 51:2401026. 10.1080/03014460.2024.2401026 39377439 10.1080/03014460.2024.2401026

[41] De Donno A, Angrisani C, Mele F, Introna F, Santoro V (2021) Dental age estimation: Demirjian's versus the other methods in different populations. A literature review. Med Sci Law 61:125–129. 10.1177/0025802420934253 33591866 10.1177/0025802420934253

[42] Sgheiza V, Liversidge HM (2023) The effect of reference sample composition and size on dental age interval estimates. Am J Biol Anthropol 182:82–92. 10.1002/ajpa.24790 37294283 10.1002/ajpa.24790

[43] Brownlee J (2018) Better deep learning: train faster, reduce overfitting, and make better predictions. Machine Learning Mastery

[44] Matthijs L, Delande L, De Tobel J et al (2024) Artificial intelligence and dental age estimation: development and validation of an automated stage allocation technique on all mandibular tooth types in panoramic radiographs. Int J Leg Med. 10.1007/s00414-024-03298-w 10.1007/s00414-024-03298-w 39105781

[45] Alkaabi S, Yussof S, Al-Mulla S (2023) Enhancing CNN for forensics age estimation using CGAN and pseudo-labelling. Comput Mater Continua 74: 2499–516. 10.32604/cmc.2023.029914

[46] Şahin TN, Kölüş T (2024) Age and sex estimation in children and young adults using panoramic radiographs with convolutional neural networks. Appl Sci 14:7014. 10.3390/app14167014

[47] Patel S (2023) Interpreting dental x-rays: A practical guide for dentists. J Oral Med 6:165. 10.35841/aaomt

[48] Demirjian A (1993) Dental development. CD-ROM ed. Silver Platter Education Montreal

[49] Levesque G-Y, Demirjian A (1980) The inter-examiner variation in rating dental formation from radiographs. J Dent Rese 59:1123–1126. 10.1177/00220345800590070401 10.1177/00220345800590070401 6929805

[50] Olze A, Bilang D, Schmidt S, Wernecke K-D, Geserick G, Schmeling A (2005) Validation of common classification systems for assessing the mineralization of third molars. Int J Leg Med 119:22–26. 10.1007/s00414-004-0489-5 10.1007/s00414-004-0489-5 15538611

[51] Dhanjal KS, Bhardwaj MK, Liversidge HM (2006) Reproducibility of radiographic stage assessment of third molars. Forensic Sci Int 159:S74–SS7. 10.1016/j.forsciint.2006.02.020 16530998 10.1016/j.forsciint.2006.02.020

[52] Konigsberg LW, Frankenberg SR, Liversidge HM (2019) Status of mandibular third molar development as evidence in legal age threshold cases. J Forensic Sci 64:680–697. 10.1111/1556-4029.13926 30296339 10.1111/1556-4029.13926

[53] Konigsberg LW, Frankenberg SR, Sgheiza V, Liversidge HM (2021) Prior probabilities and the age threshold problem: first and second molar development. Hum Biol 93:51–63. 10.13110/humanbiology.93.1.02 35338702 10.13110/humanbiology.93.1.02

[54] Tangmose S, Thevissen P, Lynnerup N, Willems G, Boldsen J (2015) Age estimation in the living: transition analysis on developing third molars. Forensic Sci Int 257:512.e1–.e7. 10.1016/j.forsciint.2015.07.049 10.1016/j.forsciint.2015.07.049 26342939

[55] Prince DA, Konigsberg LW (2008) New formulae for estimating age-at-death in the Balkans utilizing Lamendin's dental technique and Bayesian analysis. J For Sci 53:578–587. 10.1111/j.1556-4029.2008.00713.x 10.1111/j.1556-4029.2008.00713.x 18471200

[56] Lucy D, Aykroyd RG, Pollard AM, Solheim T (1996) A Bayesian approach to adult human age estimation from dental observations by johanson's age changes. J For Sci 41:15411J–J. 10.1520/jfs15411j 8871375

[57] Boldsen JL, Milner GR, Konigsberg LW, Wood JW (2002) Transition analysis: a new method for estimating age from skeletons. In: Hoppa RD, Vaupel JW (eds) Paleodemography: age distributions from skeletal samples. Cambridge University Press, Cambridge, pp 73–106

[58] Liversidge HM (2010) interpreting group differences using demirjian's dental maturity method. Forensic Science International 201:95–101 20304571 10.1016/j.forsciint.2010.02.032

[59] Demirjian A, Goldstein H, Tanner J (1973) A new system of dental age assessment. Human Biol 45:211–227 4714564

[60] Stull KE, Chu EY, Corron LK, Price MH (2023) Mixed cumulative probit: a multivariate generalization of transition analysis that accommodates variation in the shape, spread and structure of data. R Soc Open Sci 10:220963. 10.1098/rsos.220963 36866077 10.1098/rsos.220963 PMC9974299

[61] Konigsberg LW, Herrmann NP, Wescott DJ, Kimmerle EH (2008) Estimation and evidence in forensic anthropology: age-at-death. J For Sci 53:541–557. 10.1111/j.1556-4029.2008.00710.x 10.1111/j.1556-4029.2008.00710.x 18471197

[62] Konigsberg LW, Frankenberg SR (2002) Deconstructing death in paleodemography. Am J Phys Anthropol 117:297–309. 10.1002/ajpa.10039 11920365 10.1002/ajpa.10039

[63] Baylis S, Dipnall JF, Bassed R (2024) Estimating dental age of New Zealand juveniles and subadults using Demirjian's method. Forensic Sci Med Pathol 20:1343–1359. 10.1007/s12024-024-00803-w 38568351 10.1007/s12024-024-00803-w

[64] Konigsberg LW (2015) Multivariate cumulative probit for age estimation using ordinal categorical data. Ann Human Biol 42:368–378. 10.3109/03014460.2015.1045430 26190374 10.3109/03014460.2015.1045430

[65] Sivri MB, Taheri S, Kırzıoğlu Ercan RG, Yağcı Ü, Golrizkhatami Z (2024) Dental age estimation: a comparative study of convolutional neural network and Demirjian's method. J Forensic Leg Med:103. 10.1016/j.jflm.2024.102679 10.1016/j.jflm.2024.102679 38537363

[66] Covidence 2025 ed. Veritas health Innovation Melbourne, Australia

[67] Milani OH, Atici SF, Allareddy V et al (2024) A fully automated classification of third molar development stages using deep learning. Sci Rep 14:13082. 10.1038/s41598-024-63744-y 38844566 10.1038/s41598-024-63744-y PMC11156840

[68] Ong SH, Kim H, Song JS et al (2024) Fully automated deep learning approach to dental development assessment in panoramic radiographs. BMC Oral Health 24:426. 10.1186/s12903-024-04160-6 38582843 10.1186/s12903-024-04160-6 PMC10998373

[69] Costa J, Montero J, Serrano S, Albaladejo A, López-Valverde A, Bica I (2014) Accuracy in the legal age estimation according to the third molars mineralization among Mexicans and Columbians. Atencion Primaria 46:165–175. 10.1016/S0212-6567(14)70086-1 25476056 10.1016/S0212-6567(14)70086-1 PMC8171475

[70] Ong SH, Kim H, Song JS et al (2024) Fully automated deep learning approach to dental development assessment in panoramic radiographs. BMC Oral Health 24:426. 10.1186/s12903-024-04160-6 10.1186/s12903-024-04160-6 PMC10998373 38582843

[71] Mohammad N, Muad AM, Ahmad R, Mohd Yusof MYP (2021) Reclassification of Demirjian's mandibular premolars staging for age estimation based on semi-automated segmentation of deep convolutional neural network. Forensic. Imaging 24:200440. 10.1016/j.fri.2021.200440

[72] Zeng Z, Cheng X, Feng C et al (2025) Feasibility study on optimising the efficacy of a population age estimation model for south china by combined machine learning for the second and third molars. J Digit Imaging Inform Med 38:3134–3147 . 10.1007/s10278-024-01382-6 10.1007/s10278-024-01382-6 PMC12572451 39762548

[73] Mohd Yusof MYP, Wan Mokhtar I, Rajasekharan S, Overholser R, Martens L (2017) Performance of Willem’s dental age estimation method in children: a systematic review and meta-analysis. For Sci Int 280:245.e1–.e10. 10.1016/j.forsciint.2017.08.032 10.1016/j.forsciint.2017.08.032 28958768

[74] Sehrawat JS, Singh M (2017) Willems method of dental age estimation in children: a systematic review and meta-analysis. J Forensic Leg Med 52:122–129. 10.1016/j.jflm.2017.08.017 28918371 10.1016/j.jflm.2017.08.017

[75] Willems G, Van Olmen A, Spiessens B, Carels C (2001) Dental age estimation in Belgian children: Demirjian's technique revisited. J Forensic Sci 46:893–895 11451073

[76] Han M, Du S, Ge Y et al (2025) Correction to: With or without human interference for precise age estimation based on machine learning? Int J Legal Med 139:1997–2000. 10.1007/s00414-025-03468-4 40119010 10.1007/s00414-025-03468-4

[77] Duangto P, Iamaroon A, Prasitwattanaseree S, Mahakkanukrauh P, Janhom A (2017) New models for age estimation and assessment of their accuracy using developing mandibular third molar teeth in a Thai population. Int J Legal Med 131:559–568. 10.1007/s00414-016-1467-4 27757575 10.1007/s00414-016-1467-4

[78] Rocha LT, Ingold MS, Panzarella FK et al (2022) Applicability of Willems method for age estimation in Brazilian children: performance of multiple linear regression and artificial neural network. Egypt J Forensic Sci 12:9. 10.1186/s41935-022-00271-9

[79] Wu TJ, Tsai CL, Gao QZ, Chen YP, Kuo CF, Huang YH (2022) The application of artificial-intelligence-assisted dental age assessment in children with growth delay. J Pers Med:12(7):1158. 10.3390/jpm12071158 10.3390/jpm12071158 PMC9322373 35887655

[80] Yavuz BS, Ekmekcioglu O, Ankarali H (2025) Comparison of different dental age estimation methods with deep learning: Willems, Cameriere-European, London Atlas. Int J Legal Med 139:1661–1672. 10.1007/s00414-025-03452-y 39969569 10.1007/s00414-025-03452-y PMC12170674

[81] Hofmann E, Robold M, Proff P, Kirschneck C (2017) Age assessment based on third molar mineralisation : an epidemiological-radiological study on a Central-European population. J Orofac Orthop 78:97–111. 10.1007/s00056-016-0063-z 27896417 10.1007/s00056-016-0063-z

[82] Chaillet N, Demirjian A (2004) Dental maturity in South France: a comparison between Demirjian’s method and polynomial functions. J Forensic Sci 49(5):1059–1066. 10.1520/jfs2004037 15461110

[83] Pan J, Shen C, Yang Z et al (2021) A modified dental age assessment method for 5- to 16-year-old eastern Chinese children. Clin Oral Investig 25:3463–3474. 10.1007/s00784-020-03668-9 33420828 10.1007/s00784-020-03668-9 PMC8137609

[84] Cameriere R, De Angelis D, Ferrante L, Scarpino F, Cingolani M (2007) Age estimation in children by measurement of open apices in teeth: a European formula. Int J Legal Med 121:449–453. 10.1007/s00414-007-0179-1 17549508 10.1007/s00414-007-0179-1

[85] AlQahtani SJ, Hector MP, Liversidge HM (2010) Brief communication: the London atlas of human tooth development and eruption. Am J Phys Anthropol 142:481–490. 10.1002/ajpa.21258 20310064 10.1002/ajpa.21258

[86] Uribe SE, Hamdan MH, Valente NA et al (2025) Evaluating dental AI research papers: key considerations for editors and reviewers. J Dent 160:105867. 10.1016/j.jdent.2025.105867 40451605 10.1016/j.jdent.2025.105867

[87] Council NR (2009) Strengthening Forensic Science in the United States: a pathway forward. National Academies Press

[88] Tao J, Chen M, Wang J, Liu L, Hassanien AE, Xiao K (2018) Dental age estimation in east Asian population with least squares regression. In: The international conference on advanced machine learning technologies and applications (AMLTA2018), pp 653–60

[89] Wu TJ, Tsai CL, Gao QZ, Chen YP, Kuo CF, Huang YH (2022) The application of artificial-intelligence-assisted dental age assessment in children with growth delay. J Pers Med 12(7):1158. 10.3390/jpm12071158 10.3390/jpm12071158 PMC9322373 35887655

[90] Cunningham C, Scheuer L, Black S (2016) Dentition. In: Developmental Juvenile Osteology, pp 149–176

[91] Abdul Rahim AH, Davies JA, Liversidge HM (2023) Reliability and limitations of permanent tooth staging techniques. Forensic Sci Int 346:111654. 10.1016/j.forsciint.2023.111654 37011430 10.1016/j.forsciint.2023.111654

[92] Kapoor P, Jain V (2018) Comprehensive chart for dental age estimation (DAEcc8) based on Demirjian 8-teeth method: simplified for operator ease. J Forensic Leg Med 59:45–49 30138901 10.1016/j.jflm.2018.07.014

[93] Melo M, Ata-Ali J (2017) Accuracy of the estimation of dental age in comparison with chronological age in a Spanish sample of 2641 living subjects using the Demirjian and Nolla methods. Forensic Sci Int 270:276.e1–.e7. 10.1016/j.forsciint.2016.10.001 10.1016/j.forsciint.2016.10.001 28029496

[94] Chaillet N, Nyström M, Demirjian A (2005) Comparison of dental maturity in children of different ethnic origins: international maturity curves for clinicians. J Forensic Sci 50:JFS2005020–11. 10.1520/jfs2005020 16225225

[95] Jayaraman J, Wong HM, King NM, Roberts GJ (2013) The French-Canadian data set of Demirjian for dental age estimation: a systematic review and meta-analysis. J Forensic Leg Med 20:373–381. 10.1016/j.jflm.2013.03.015 23756500 10.1016/j.jflm.2013.03.015

