# Supplementary material for: Dental age estimation: a scoping review comparing the manual application of the Demirjian method and artificial intelligence modalities
Source: Int J Legal Med. 2026 Feb 23;140(3):1451–69. doi: 10.1007/s00414-026-03721-4 (PMC13161306; doi:10.1007/s00414-026-03721-4)
Supplement: Supplementary file 1 — Supplementary Material 1 (PDF 175 KB) [file 414_2026_3721_MOESM1_ESM.pdf]

Title: Dental age estimation: A scoping review comparing the manual application of the Demirjian method and artificial intelligence modalities.

Journal: International Journal of Forensic Medicine

Authors:
Dr. Stephanie Baylis
BDS, MForensMed, MPhil
Department of Forensic Medicine, School of Public Health and Preventive Medicine, Monash University Melbourne, Victoria, Australia
Baylis Dental Services, Whangarei, Northland, New Zealand
ORCID: 0000-0001-8697-0996
Corresponding Author: stephanie1baylis@gmail.com

Dr. Joanna F Dipnall
B.Ec (Hons), PhD
School of Public Health and Preventive Medicine, Monash University Melbourne, Victoria, Australia
Institute for Mental and Physical Health and Clinical Translation, School of Medicine Deakin University, Geelong, Australia
ORCID: 0000-0001-7543-0687

Professor Richard Bassed
BDS, PhD, DipForOdont, FFOMP (RCPA)
Victorian Institute of Forensic Medicine and the Department of Forensic Medicine, Monash University, Melbourne, Victoria, Australia
ORCID: 0000-0001-5473-055X

Online Resource 1.

Table 1. General terms – adapted from Delua (2021)

Term	Definition
**Supervised learning**	Labelled target data with inputs used to learn the data to make predictions
**Unsupervised learning**	Automatically extract features from large unstructured/unlabelled datasets to make own predictions. Also known as end-to-end
**Semi-supervised learning**	Portion of target data labelled so that combines supervised and unsupervised learning to train models for prediction tasks
**Transfer learning**	Knowledge learnt from one task or dataset is used to improve the model performance when applied to a different task or dataset
**Reinforcement learning**	Mimics real-world biological learning methods through positive reinforcement; does not produce labels or measure against ground truth

Delua J. (2021) Supervised vs unsupervised learning; What's the difference? Think topics. IBM. https://www.ibm.com/think/topics/supervised-vs-unsupervised-learning. Accessed 5 May 2025

Table 2. Machine learning algorithms in DAE accessed – adapted from Brownlee (2023)

**Parametric algorithms in DAE**	
**Regression**	Find relationships between variables in x-ray data<br>May be linear, multiple, Bayesian, logistic, polynomial
**Non-parametric algorithms in DAE**	
**Decision tree (DT)**	Interconnected decisions for age classification & predictions
**Random forest (RF)**	Uses multiple decision trees with majority voting
**K-nearest neighbour (K-NN)**	Supervised; Predicts age based on proximity of data in x-ray
**Support Vector Machine (SVM)**	Supervised; Divides complex data in multidimensional space; converts nonlinear problems into linear ones
**AdaBoost**	Iterative error correction and data weighting
**Gradient Boosting**	Iterative error correction and data weighting
**Multilayer Perceptrons (MLP)**	Learn from nonlinear relationships in data; classification, regression, pattern recognition; process layered information

Brownlee J. (2023) A tour of machine learning algorithms. Machine Learning Algorithms. https://machinelearningmastery.com/a-tour-of-machine-learning-algorithms/ Accessed 8 May 2025
