# Supplementary material for: Dental age estimation: a scoping review comparing the manual application of the Demirjian method and artificial intelligence modalities
Source: Int J Legal Med. 2026 Feb 23;140(3):1451–69. doi: 10.1007/s00414-026-03721-4 (PMC13161306; doi:10.1007/s00414-026-03721-4)
Supplement: Supplementary file 2 — Supplementary Material 2 (PDF 209 KB) [file 414_2026_3721_MOESM2_ESM.pdf]

Title: Dental age estimation: A scoping review comparing the manual application of the Demirjian method and artificial intelligence modalities.

Journal: International Journal of Forensic Medicine

Authors:
Dr. Stephanie Baylis
BDS, MForensMed, MPhil
Department of Forensic Medicine, School of Public Health and Preventive Medicine, Monash University Melbourne, Victoria, Australia
Baylis Dental Services, Whangarei, Northland, New Zealand
ORCID: 0000-0001-8697-0996
Corresponding Author: stephanie1baylis@gmail.com

Dr. Joanna F Dipnall
B.Ec (Hons), PhD
School of Public Health and Preventive Medicine, Monash University Melbourne, Victoria, Australia
Institute for Mental and Physical Health and Clinical Translation, School of Medicine Deakin University, Geelong, Australia
ORCID: 0000-0001-7543-0687

Professor Richard Bassed
BDS, PhD, DipForOdont, FFOMP (RCPA)
Victorian Institute of Forensic Medicine and the Department of Forensic Medicine, Monash University, Melbourne, Victoria, Australia
ORCID: 0000-0001-5473-055X

Online Resource 3.

Table 1 Comparison of De Tobel modified Demirjian stages and the 1973 Demirjian stages

Demirjian Stages	O-C (0-3)	D (4)	E (5)	F (6)	G (7)	N/A	H (8)
De Tobel Stages	(0-3)	(4)	(5)	(6)	(7)	(8)	(9)
Demirjian	No change	a) b) - No change<br>**c) Beginning of root formation is seen in the form of a spicule**	*a) Initial formation of the radicular bifurcation is seen in the form of either a calcified point or a semi-lunar shape* [a]<br>*b) The root length is still less than the crown height* [b]	No change	No change		No change [c]
De Tobel	No change	a) b) - No change	**a) Beginning of root formation is seen in the form of a spicule**<br>*b) The root length is still less than the crown height* [a]<br>*c) Initial formation of the radicular bifurcation is seen in the form of either a calcified point or a semi-lunar shape*	No change	No change	**The walls of the root canal are now converging at the apex. The apical end is still partially open**	No change [c]

BOLD text indicates change in stage criteria; *Italic* text indicates change in criteria order (relevant to the Demirjian method rules)
[a] Description for uniradicular teeth removed
[b] not updated to 1980 version
[c] No change means stage criteria match between methods

De Tobel J, Radesh P, Vandermeulen D, Thevissen PW (2017) An automated technique to stage lower third molar development on panoramic radiographs for age estimation: a pilot study. The Journal of forensic odonto-stomatology 2: 49–60.

Table 2. Comparison of the Milani modified Demirjian stages and the 1973 Demirjian stages

Demirjian Stages		O	A (1)	B (2)	C (3)	D	E	F	G	H
Demirjian		No calcification		Fusion of the calcified points forms one or several cusps uniting to give a regularly outlined occlusal surface	a) Enamel formation is complete at the occlusal surface: extension and convergence towards the cervical region is seen<br>b) **The beginning of a dentinal deposit is seen**<br>c) The outline of the pulp chamber has a curved shape at the occlusal border	a) **The crown formation is completed down to the CEJ**<br>***b) In molars the pulp chamber has a trapezoidal form.***<br>c) Beginning of root formation is seen in the form of a spicule	**a) Initial formation of the radicular bifurcation is seen in the form of either a calcified point or a semi-lunar shape.**<br>***b) The root length is less than the crown height***	a) The walls of the pulp chamber now form a, more or less, isosceles triangle. **The apex ends in a funnel shape**<br>b) ***The root length is equal to or greater than the crown height***	**The walls of the root canal are now parallel** and its apical end is still partially open	No change [a]
Milani Stages	1	2	3 (A)	4 (B)	5 (C)	6 (D)	7 (E)	8 (F)	9 (G)	10 (H)
Milani	Missing lower 3rd molar	Empty follicle	Cusp tips are mineralized but have not yet coalesced	Mineralized cusps are united so the mature coronal morphology is well-defined	The crown is about half formed [b]; the pulp chamber is evident and **dentinal deposition is occurring.**	**Crown formation is complete to the dentinoenamel junction.** ***The pulp chamber has a trapezoidal form***	**Formation of the inter-radicular bifurcation has begun.** ***Root length is less than the crown length***	***Root length is at least as great as crown length.*** **Roots have funnel-shaped endings**	**Root walls are parallel,** but apices remain open	No change [a]

BOLD, ***BOLD and Italic,*** *underlined and italic* text used highlights matching criteria between the Demirjian and the Milani stages

[a] No change means the stage criteria match between methods

[b] The Demirjian method does not use absolute measures compared to this stage definition by Milani

Milani OH, Atici SF, Allareddy V et al (2024) A fully automated classification of third molar development stages using deep learning. Sci Rep 14: 13082. doi: 10.1038/s41598-024-63744-y

Table 3 Comparison of the Mohammad modified Demirjian stages and the 1973 Demirjian stages

Demirjian Stages	B	C	D	E		F	G	H
Demirjian	Fusion of the calcified points forms one or several cusps uniting to give a regularly outlined occlusal surface	a) **Enamel formation is complete at the occlusal surface**: extension and convergence towards the cervical region is seen<br>b) ***The beginning of a dentinal deposit is seen***<br>c) The outline of the pulp chamber has a curved shape at the occlusal border	a) **The crown formation is completed down to the CEJ**<br>b) The superior border of the *pulp chamber in the uniradicular teeth has a definite curved form, being concave towards the cervical region.* The projection of the pulp horns if present, gives an outline shaped like an umbrella top<br>c) Beginning of ***root formation is seen*** in the form of a spicule	a) **The walls of the pulp chamber now form straight** lines, whose continuity is broken by the presence of the pulp horn, which is larger than the previous stage<br>b) ***The root length is less than the crown height***		a) The walls of the pulp chamber now form a, more or less, isosceles triangle. **The apex ends in a funnel shape**<br>b) ***The root length is equal to or greater than the crown height***	**The walls of the root canal are now parallel and its apical end is still partially open**	**a) The apical end of the root canal is completely closed**<br>b) The periodontal membrane has a uniform width around the root and apex
Mohammad Stages	C	D1	D2	D3	D4	D5	E	F
Mohammad	Calcified cusps are united, so an outlined occlusal surface is well defined	**Enamel formation has been completed at the occlusal surface** and ***the dentine formation has started***	**The crown formation is complete to the CEJ** and ***root formation is seen.*** *The pulp chamber is being concave toward the cervical region*	**The walls of the pulp chamber are straight** and the ***root length is less than the crown height***	**The walls of the pulp chamber are straight,** where 2/3 of the root has been developed [a]	**Apex ends in a funnel shape** and ***the root length is equal to or greater than the crown***	**The walls of the root canal are now parallel and its apical end is partially open**	**The apical end of the root canal is completely closed**

BOLD, ***BOLD and Italic,*** *underlined and italic* text used highlights matching criteria between the Demirjian and the Mohammad stages

[a] The Demirjian method does not use absolute measures compared to this stage definition by Mohammad

Mohammad N, Muad AM, Ahmad R, Mohd Yusof MYP (2021) Reclassification of Demirjian's mandibular premolars staging for age estimation based on semi-automated segmentation of deep convolutional neural network. Forensic Imaging 24: 200440–. doi: 10.1016/j.fri.2021.200440
