# Supplementary material for: Dental age estimation: a scoping review comparing the manual application of the Demirjian method and artificial intelligence modalities
Source: Int J Legal Med. 2026 Feb 23;140(3):1451–69. doi: 10.1007/s00414-026-03721-4 (PMC13161306; doi:10.1007/s00414-026-03721-4)
Supplement: Supplementary file 3 — Supplementary Material 3 (PDF 144 KB) [file 414_2026_3721_MOESM3_ESM.pdf]

Title: Dental age estimation: A scoping review comparing the manual application of the Demirjian method and artificial intelligence modalities.
Journal: International Journal of Forensic Medicine

Authors:
Dr. Stephanie Baylis
BDS, MForensMed, MPhil
Department of Forensic Medicine, School of Public Health and Preventive Medicine, Monash University Melbourne, Victoria, Australia
Baylis Dental Services, Whangarei, Northland, New Zealand
ORCID: 0000-0001-8697-0996
Corresponding Author: stephanie1baylis@gmail.com

Dr. Joanna F Dipnall
B.Ec (Hons), PhD
School of Public Health and Preventive Medicine, Monash University Melbourne, Victoria, Australia
Institute for Mental and Physical Health and Clinical Translation, School of Medicine Deakin University, Geelong, Australia
ORCID: 0000-0001-7543-0687

Professor Richard Bassed
BDS, PhD, DipForOdont, FFOMP (RCPA)
Victorian Institute of Forensic Medicine and the Department of Forensic Medicine, Monash University, Melbourne, Victoria, Australia
ORCID: 0000-0001-5473-055X

Online Resource 3

Literature Search

Search strategies were drafted by SB and refined through team discussion. With the assistance of a medical librarian, the following seven scholarly literature databases were searched on the 5th September 2024 and again; Medline (Ovid Platform), Embase (Ovid Platform), Global Health (Ovid Platform), Scopus (Elsevier Platform), Social Science Premium Collection (Proquest Platform), CENTRAL-Cochrane Library Central Register of Controlled Trials (Wiley Platform), and NZLII-New Zealand Legal Information Institute (NZLII Website). A combination of subject headings and keywords were searched using the Boolean Operator OR, covering the main concepts of DAE and ML. These two concepts were then combined with the Boolean Operator AND. No limits or filters were applied, and each database was searched from their inception date. A re-run of all databases to capture recent references was conducted on the 9thApril 2025, with the publication limit of 5th September 2024-onwards placed for all databases. A detailed search strategy for each database, listing all subject heading and keywords used for this search were as follows:

Medline:

1	exp Age Determination by Teeth/
2	(dental adj2 age).ti,ab.
3	(tooth adj2 age).ti,ab.
4	(teeth adj2 age).ti,ab.
5	(incisor* adj2 age).ti,ab.
6	(canine* adj2 age).ti,ab.
7	(premolar* adj2 age).ti,ab.
8	(molar* adj2 age).ti,ab.
9	(mandib* adj2 age).ti,ab.
10	(jaw bone* adj2 age).ti,ab.
11	(jawbone* adj2 age).ti,ab.
12	1 or 2 or 3 or 4 or 5 or 6 or 7 or 8 or 9 or 10 or 11
13	age estimat*.ti,ab.
14	age assess*.ti,ab.
15	age determinat*.ti,ab.
16	age regress*.ti,ab.
17	13 or 14 or 15 or 16

18 exp Tooth/
19 exp Forensic Dentistry/
20 exp Dental Care/
21 exp Dental Research/
22 exp Dental Caries/
23 exp Dental Implants/
24 exp Dental Clinics/
25 exp Technology, Dental/
26 exp Societies, Dental/
27 exp Dentist/
28 exp Orthodontics/
29 exp Orthodontics, Corrective/
30 tooth.ti,ab.
31 teeth.ti,ab.
32 canine*.ti,ab.
33 incisor*.ti,ab.
34 premolar*.ti,ab.
35 molar*.ti,ab.
36 mandib*.ti,ab.
37 jaw bone*.ti,ab.
38 jawbone*.ti,ab.
39 dental.ti,ab.
40 dentist*.ti,ab.
41 orthodont*.ti,ab.
42 18 or 19 or 20 or 21 or 22 or 23 or 24 or 25 or 26 or 27 or 28 or 29 or 30 or 31 or 32 or 33 or 34 or 35 or 36 or 37 or 38 or 39 or 40 or 41
43 17 and 42
44 12 or 43
45 exp Machine Learning/
46 exp Artificial Intelligence/
47 exp Pattern Recognition, Automated/
48 exp Algorithms/
49 exp Neural Networks, Computer/
50 machine learning.ti,ab.
51 artificial intelligence.ti,ab.
52 neural network*.ti,ab.
53 pattern recognition.ti,ab.
54 algorithm*.ti,ab.
55 large language model*.ti,ab.
56 deep learning*.ti,ab.
57 supervised learning*.ti,ab.
58 unsupervised learning*.ti,ab.
59 (semi supervised learning* or semi-supervised learning*).ti,ab.
60 reinforcement learning*.ti,ab.
61 transfer learning*.ti,ab.
62 (convolutional adj2 network*).ti,ab.
63 45 or 46 or 47 or 48 or 49 or 50 or 51 or 52 or 53 or 54 or 55 or 56 or 57 or 58 or 59 or 60 or 61 or 62
64 44 and 63

Embase:
1 exp Dental Age Estimation/
2 (dental adj2 age).ti,ab.
3 (tooth adj2 age).ti,ab.
4 (teeth adj2 age).ti,ab.
5 (incisor* adj2 age).ti,ab.
6 (canine* adj2 age).ti,ab.
7 (premolar* adj2 age).ti,ab.
8 (molar* adj2 age).ti,ab.
9 (mandib* adj2 age).ti,ab.
10 (jaw bone* adj2 age).ti,ab.

11	(jawbone* adj2 age).ti,ab.
12	1 or 2 or 3 or 4 or 5 or 6 or 7 or 8 or 9 or 10 or 11
13	exp Age Determination/
14	age estimat*.ti,ab.
15	age assess*.ti,ab.
16	age determinat*.ti,ab.
17	age regress*.ti,ab.
18	13 or 14 or 15 or 16 or 17
19	exp Tooth/
20	exp Forensic Odontology/
21	exp Dental Procedure/
22	exp Dental Research/
23	exp Dental Caries/
24	exp Tooth Implants/
25	exp Dental Clinic/
26	exp Dental Technology/
27	exp Dentist/
28	exp Pediatric Dentist/
29	exp Orthodontics/
30	tooth.ti,ab.
31	teeth.ti,ab.
32	canine*.ti,ab.
33	incisor*.ti,ab.
34	premolar*.ti,ab.
35	molar*.ti,ab.
36	mandib*.ti,ab.
37	jaw bone*.ti,ab.
38	jawbone*.ti,ab.
39	dental.ti,ab.
40	dentist*.ti,ab.
41	orthodont*.ti,ab.
42	19 or 20 or 21 or 22 or 23 or 24 or 25 or 26 or 27 or 28 or 29 or 30 or 31 or 32 or 33 or 34 or 35 or 36 or 37 or 38 or 39 or 40 or 41
43	18 and 42
44	12 or 43
45	exp Machine Learning/
46	exp Supervised Machine Learning/
47	exp Unsupervised Machine Learning/
48	exp "Reinforcement Learning (Machine Learning)"/
49	exp Artificial Intelligence/
50	exp Automated Pattern Recognition/
51	exp Algorithm/
52	exp Artificial Neural Network/
53	exp Large Language Model/
54	exp Deep Learning/
55	exp Convolutional Neural Network/
56	machine learning.ti,ab.
57	artificial intelligence.ti,ab.
58	neural network*.ti,ab.
59	pattern recognition.ti,ab.
60	algorithm*.ti,ab.
61	large language model*.ti,ab.
62	deep learning*.ti,ab.
63	supervised learning*.ti,ab.
64	unsupervised learning*.ti,ab.
65	(semi supervised learning* or semi-supervised learning*).ti,ab.
66	reinforcement learning*.ti,ab.
67	transfer learning*.ti,ab.
68	(convolutional adj2 network*).ti,ab.
69	45 or 46 or 47 or 48 or 49 or 50 or 51 or 52 or 53 or 54 or 55 or 56 or 57 or 58 or 59 or 60 or 61 or 62

or 63 or 64 or 65 or 66 or 67 or 68
70 44 and 69

Global Health:
SearchesResults
1 (dental adj2 age).ti,ab.
2 (tooth adj2 age).ti,ab.
3 (teeth adj2 age).ti,ab.
4 (incisor* adj2 age).ti,ab.
5 (canine* adj2 age).ti,ab.
6 (premolar* adj2 age).ti,ab.
7 (molar* adj2 age).ti,ab.
8 (mandib* adj2 age).ti,ab.
9 (jaw bone* adj2 age).ti,ab.
10 (jawbone* adj2 age).ti,ab.
11 1 or 2 or 3 or 4 or 5 or 6 or 7 or 8 or 9 or 10
12 exp Age Determination/
13 age estimat*.ti,ab.
14 age assess*.ti,ab.
15 age determinat*.ti,ab.
16 age regress*.ti,ab.
17 12 or 13 or 14 or 15 or 16
18 exp Teeth/
19 exp Dental Caries/
20 exp Dental Implants/
21 exp Dental Health/
22 exp Dentistry/
23 exp Dentists/
24 exp Tooth Diseases/
25 exp Periodontal Diseases/
26 tooth.ti,ab.
27 teeth.ti,ab.
28 canine*.ti,ab.
29 incisor*.ti,ab.
30 premolar*.ti,ab.
31 molar*.ti,ab.
32 mandib*.ti,ab.
33 jaw bone*.ti,ab.
34 jawbone*.ti,ab.
35 dental.ti,ab.
36 dentist*.ti,ab.
37 orthodont*.ti,ab.
38 18 or 19 or 20 or 21 or 22 or 23 or 24 or 25 or 26 or 27 or 28 or 29 or 30 or 31 or 32 or 33 or 34 or 35 or 36 or 37
39 17 and 38
40 11 or 39 430
41 exp Machine Learning/
42 exp Artificial Intelligence/
43 exp Neural Networks/
44 exp Algorithms/
45 machine learning.ti,ab.
46 artificial intelligence.ti,ab.
47 neural network*.ti,ab.
48 pattern recognition.ti,ab.
49 algorithm*.ti,ab.
50 large language model*.ti,ab.
51 deep learning*.ti,ab.
52 supervised learning*.ti,ab.
53 unsupervised learning*.ti,ab.
54 (semi supervised learning* or semi-supervised learning*).ti,ab.

55 reinforcement learning*.ti,ab.
56 transfer learning*.ti,ab.
57 (convolutional adj2 network*).ti,ab.
58 41 or 42 or 43 or 44 or 45 or 46 or 47 or 48 or 49 or 50 or 51 or 52 or 53 or 54 or 55 or 56 or 57
59 40 and 58

Scopus:
((TITLE-ABS (convolutional W/2 network*)) OR (TITLE-ABS ("transfer learning*")) OR (TITLE-ABS ("reinforcement learning*")) OR (TITLE-ABS ("semi supervised learning*" OR "semi-supervised learning*")) OR (TITLE-ABS ("unsupervised learning*")) OR (TITLE-ABS ("supervised learning*")) OR (TITLE-ABS ("deep learning*")) OR (TITLE-ABS ("large language model*")) OR (TITLE-ABS (algorithm*)) OR (TITLE-ABS ("pattern recognition")) OR (TITLE-ABS ("neural network*")) OR (TITLE-ABS ("artificial intelligence")) OR (TITLE-ABS ("machine learning")) OR (INDEXTERMS ("Neural Networks, Computer")) OR (INDEXTERMS (algorithms)) OR (INDEXTERMS ("Pattern Recognition, Automated")) OR (INDEXTERMS ("Artificial Intelligence")) OR (INDEXTERMS ("Machine Learning"))) AND ((((TITLE-ABS (orthodont*)) OR (TITLE-ABS (dentist*)) OR (TITLE-ABS (dental)) OR (TITLE-ABS (jawbone*)) OR (TITLE-ABS ("jaw bone*")) OR (TITLE-ABS (mandib*)) OR (TITLE-ABS (molar*)) OR (TITLE-ABS (premolar*)) OR (TITLE-ABS (incisor*)) OR (TITLE-ABS (canine*)) OR (TITLE-ABS (teeth)) OR (TITLE-ABS (tooth)) OR (INDEXTERMS ("Orthodontics, Corrective")) OR (INDEXTERMS (orthodontics)) OR (INDEXTERMS (dentist)) OR (INDEXTERMS ("Societies, Dental")) OR (INDEXTERMS ("Technology, Dental")) OR (INDEXTERMS ("Dental Clinics")) OR (INDEXTERMS ("Dental Implants")) OR (INDEXTERMS ("Dental Caries")) OR (INDEXTERMS ("Dental Research")) OR (INDEXTERMS ("Dental Care")) OR (INDEXTERMS ("Forensic Dentistry")) OR (INDEXTERMS (tooth))) AND ((TITLE-ABS ("age assess*")) OR (TITLE-ABS ("age regress*")) OR (TITLE-ABS ("age determinat*")) OR (TITLE-ABS ("age estimat*")))) OR ((TITLE-ABS (jawbone* W/2 age)) OR (TITLE-ABS ("jaw bone*" W/2 age)) OR (TITLE-ABS (mandib* W/2 age)) OR (TITLE-ABS (molar* W/2 age)) OR (TITLE-ABS (premolar* W/2 age)) OR (TITLE-ABS (canine* W/2 age)) OR (TITLE-ABS (incisor* W/2 age)) OR (TITLE-ABS (teeth W/2 age)) OR (TITLE-ABS (tooth W/2 age)) OR (TITLE-ABS (dental W/2 age)) OR (INDEXTERMS ("Age Determination by Teeth")))))

Social Science Premium Collection:
S7 [S5] AND [S6]
S6 TI,AB("machine learning") OR TI,AB("artificial intelligence") OR TI,AB(("neural network" OR "neural networks")) OR TI,AB("pattern recognition") OR TI,AB(algorithm*) OR TI,AB("large language model*") OR TI,AB(("deep learning")) OR TI,AB(("supervised learning")) OR TI,AB(("unsupervised learning")) OR (TI,AB("semi supervised learning*") OR TI,AB("semi-supervised learning*")) OR TI,AB(("reinforcement learning")) OR TI,AB("transfer learning*") OR (TI,AB(convolutional) NEAR/2 TI,AB(network*))
S5 [S1] OR [S4]
S4 [S2] AND [S3]
S3 TI,AB(tooth) OR TI,AB(teeth) OR TI,AB(canine*) OR TI,AB(incisor*) OR TI,AB(premolar*) OR TI,AB(molar*) OR TI,AB(mandib*) OR TI,AB("jaw bone*") OR TI,AB(jawbone*) OR TI,AB(dentist*) OR TI,AB(dental) OR TI,AB(orthodont*)
S2 TI,AB("age estimat*") OR TI,AB("age assess*") OR TI,AB("age determinat*") OR (TI,AB(incisor*) NEAR/2 TI,AB(age))
S1 (TI,AB(dental) NEAR/2 TI,AB(age)) OR (TI,AB(tooth) NEAR/2 TI,AB(age)) OR (TI,AB(teeth) NEAR/2 TI,AB(age)) OR (TI,AB(incisor*) NEAR/2 TI,AB(age)) OR (TI,AB(canine*) NEAR/2 TI,AB(age)) OR (TI,AB(premolar*) NEAR/2 TI,AB(age)) OR (TI,AB(molar*) NEAR/2 TI,AB(age)) OR (TI,AB(mandib*) NEAR/2 TI,AB(age)) OR (TI,AB(jawbone*) NEAR/2 TI,AB(age))

CENTRAL:
#1 [mh "Age Determination by Teeth"]
#2 (dental:ti,ab NEAR/2 age:ti,ab)
#3 (tooth:ti,ab NEAR/2 age:ti,ab)
#4 (teeth:ti,ab NEAR/2 age:ti,ab)
#5 (incisor*:ti,ab NEAR/2 age:ti,ab)
#6 (canine*:ti,ab NEAR/2 age:ti,ab)
#7 (premolar*:ti,ab NEAR/2 age:ti,ab)
#8 (molar*:ti,ab NEAR/2 age:ti,ab)
#9 (mandib*:ti,ab NEAR/2 age:ti,ab)

#10 (("jaw" NEXT bone*):ti,ab NEAR/2 age:ti,ab)
#11 (jawbone*:ti,ab NEAR/2 age:ti,ab)
#12 #1 OR #2 OR #3 OR #4 OR #5 OR #6 OR #7 OR #8 OR #9 OR #10 OR #11
#13 ("age" NEXT estimat*):ti,ab
#14 ("age" NEXT assess*):ti,ab
#15 ("age" NEXT determinat*):ti,ab
#16 ("age" NEXT regress*):ti,ab
#17 #13 OR #14 OR #15 OR #16
#18 [mh Tooth]
#19 [mh "Forensic Dentistry"]
#20 [mh "Dental Care"]
#21 [mh "Dental Research"]
#22 [mh "Dental Caries"]
#23 [mh "Dental Implants"]
#24 [mh "Dental Clinics"]
#25 [mh "Technology, Dental"]
#26 [mh "Societies, Dental"]
#27 [mh Dentist]
#28 [mh Orthodontics]
#29 [mh "Orthodontics, Corrective"]
#30 tooth:ti,ab
#31 teeth:ti,ab
#32 canine*:ti,ab
#33 incisor*:ti,ab
#34 premolar*:ti,ab
#35 molar*:ti,ab
#36 mandib*:ti,ab
#37 ("jaw" NEXT bone*):ti,ab
#38 jawbone*:ti,ab
#39 dental:ti,ab
#40 dentist*:ti,ab
#41 orthodont*:ti,ab
#42 #18 OR #19 OR #20 OR #21 OR #22 OR #23 OR #24 OR #25 OR #26 OR #27 OR #28 OR #29 OR #30 OR #31 OR #32 OR #33 OR #34 OR #35 OR #36 OR #37 OR #38 OR #39 OR #40 OR #41
#43 #17 AND #42
#44 #12 OR #43
#45 [mh "Machine Learning"]
#46 [mh "Artificial Intelligence"]
#47 [mh "Pattern Recognition, Automated"]
#48 [mh Algorithms]
#49 [mh "Neural Networks, Computer"]
#50 ("machine" NEXT learning):ti,ab
#51 ("artificial" NEXT intelligence):ti,ab
#52 ("neural" NEXT network*):ti,ab
#53 ("pattern" NEXT recognition):ti,ab
#54 algorithm*:ti,ab
#55 ("large" NEXT language NEXT model*):ti,ab
#56 ("deep" NEXT learning*):ti,ab
#57 ("supervised" NEXT learning*):ti,ab
#58 ("unsupervised" NEXT learning*):ti,ab
#59 (("semi" NEXT supervised NEXT learning*):ti,ab OR ("semi-supervised" NEXT learning*):ti,ab)
#60 ("reinforcement" NEXT learning*):ti,ab
#61 ("transfer" NEXT learning*):ti,ab
#62 (convolutional:ti,ab NEAR/2 network*:ti,ab)
#63 #45 OR #46 OR #47 OR #48 OR #49 OR #50 OR #51 OR #52 OR #53 OR #54 OR #55 OR #56 OR #57 OR #58 OR #59 OR #60 OR #61 OR #62
#64 #44 AND #63

NZLII (New Zealand Legal Information):
dental age estimation and machine learning

dental age estimation and artificial intelligence
dental age estimation and neural network
dental age estimation and large language model
dental age determination and machine learning
dental age determination and artificial intelligence
dental age determination and neural network
dental age determination and large language model
machine learning and teeth
artificial intelligence and teeth
neural network and teeth
large language model and teeth
machine learning and dental
artificial intelligence and dental
neural network and dental
large language model and dental

Eligibility criteria were set for the screening process as: not Demirjian/modified Demirjian studies; not OPG/Panoramic; not AI/ML/DL; not DAE; non-dental feature/s; not human. Exclusion criteria for full-text studies included sample bias, unclear sample distribution, comparator bias/exclusion, missing results, unclear methodology, indirect comparisons, irrelevant methodology, multiple papers with sample methodology by one research team. The following inclusion criteria were considered eligible: quantitative research articles; application of AI/ML/DL involving the Demirjian/modified Demirjian techniques; available in English language; full-text studies; OPG/Panoramic; study age-ranges included children/juveniles/subadults. References cited in the selected studies and any systematic reviews found, were reviewed for further relevant studies. One study correction was sought. A search of Google and Google Scholar using the same keywords covering the main concepts of DAE and ML, was undertaken to locate any further relevant grey literature, on the 26thApril 2025.

The final search results were exported into Covidence Systematic Review software for removal of duplicates and undertaking of the screening process. Two reviewers (SB and AF) screened titles and abstracts, within Covidence. Any conflicts were discussed and resolved with two other reviewers (JD and RB). Full text reading was carried out by the SB with support JD and RB.

The first search yielded 595 articles of which 265 were duplicates. From the yield of 330, 20 full text articles were sourced. A yield of 71 came from the second search, of which 30 were duplicates. The yield of 41 articles came from the second search, of which two full-text articles were sourced, one of which was a correction for an article sourced in the first search. A final total of 22 articles from an initial total of 666 articles was used in this scoping review. The corrected article was combined with the original article as a count of one.
